# The Variability of Mental Timeline in Vertical Dimension

**DOI:** 10.3389/fpsyg.2021.782975

**Published:** 2021-12-31

**Authors:** Jiaoyan He, Cuihua Bi, Hao Jiang, Jianan Meng

**Affiliations:** Faculty of Psychology, Sichuan Normal University, Chengdu, China

**Keywords:** vertical dimension, mental timeline, visual experience, variability, implicit and explicit

## Abstract

People often use concrete spatial terms to represent abstract time. Previous studies have shown that mental timeline (MTL) is represented along a horizontal axis. Studies of the mental timeline have demonstrated that compared with English speakers, Mandarin speakers are more likely to think about time vertically (up-down) than horizontally (left-right/front-back). Prior studies have suggested that MTL in the up and down dimensions originated from temporal-spatial metaphors in language. However, there are still a large number of perceptual experiences in the up and down dimensions, such as visual and sensorimotor experience. Then does the visual experience in daily life affect the MTL in the vertical dimension? This study is aimed to investigate whether visual experience can promote or activate the opposite direction of MTL from implicit and explicit levels. The results showed that when the time information in the task was not prominent, the direction of vertical MTL cannot be affected by ascending or descending perceptual experience. While when the time information was prominent, whether the task was implicit or explicit, compared with the control group, watching the top-down scene significantly increased the top-down direction selection, while in the implicit task, watching the bottom-up scene made the top-down MTL disappear. To the best of our knowledge, our study provides the first evidence that the flexibility of space–time associations in vertical dimension extends beyond explicit and embraces even implicit levels. This study shows that the vertical MTL is activated in certain conditions and could be affected by the visual experience.

## Introduction

The concept of time and space are indispensable for our thinking. The connection between time and space not only exists in language expression but also in the cognitive processing of information. A large number of studies have shown that time is represented by means of space (Santiago et al., 2007; Weger and Pratt, 2008). The connection between time and space is manifested in length, distance, direction, and other aspects. In terms of direction, people tend to imagine time from past to future as a line extending along a certain spatial direction, namely the mental timeline (MTL). The spatial representation of time is multi-directional: in the left and right dimensions, past/short time/early stimuli are represented on the left side of the timeline, while future/long time/late stimuli are represented on the right side of the timeline (Vallesi et al., 2011; Eikmeier et al., 2015). In the front and back dimensions, people tend to put the future time in front and the past time behind. In the up and down dimensions, “up” is associated with past temporal stimuli, and “down” is associated with future temporal stimuli (de la Fuente et al., 2014; von Sobbe et al., 2019).

The MTL has been confirmed by many studies in the left-right and front-back axis. Santiago et al. (2007) presented some words referring either to the past or to the future, and asked the subjects to press the left and right keys to classify them. The results showed that the subjects responded faster when they used the left key to react to the past words and the right key to react to the future words. By using different stimulating materials, such as pictures (Fuhrman and Boroditsky, 2010), video (Santiago et al., 2010), duration (Casasanto and Boroditsky, 2008; Ishihara et al., 2008; Coull et al., 2018; Isham et al., 2018), past or future time words or events (Santiago et al., 2007; Ouellet et al., 2010; Ding et al., 2015), actor names (Weger and Pratt, 2008) and auditory stimulus (Kong and You, 2012), they found that the left hand reacted more quickly to “past/short time/early events” and the right hand reacted more quickly to “future/long time/late events.” Using eye movement as an indicator, subjects’ eyes shifted to the left when thinking about the past and to the right when thinking about the future (Anelli et al., 2018; He et al., 2018).

The existing studies have done a lot of research on the left-right and front-back dimensions, but there are still many disputes about the MTL in the vertical direction. Boroditsky (2001) required the subjects to answer two spatial priming questions and then asked them to answer a time-related question. The spatial priming questions described the horizontal or vertical spatial relationship such as “the black bug crawling before the white bug (left)” and “the black ball is above the white ball.” The results showed that the Mandarin subjects had a shorter reaction time to complete the time–related task after the vertical space was activated. Subsequently, the researchers directly manipulated these stimuli, they selected images containing the time range from only a few seconds to decades of years. And then asked the subjects to judge the second picture was earlier or later than the first picture by pressing the left and right keys in the horizontal direction or the up and down keys in the vertical direction. The result showed that there was early-up and late-down temporal-spatial correlation in the vertical dimension. Compared with native English speakers, Mandarin speakers talk more about MTL in the vertical dimension. Especially when the subjects were really good at Chinese and the experimental material was in Chinese, they were more inclined to represent time in the vertical dimension (Boroditsky et al., 2011). These studies have shown that the experience of spatial metaphor has a long-term impact on the representation of time. For Mandarin Chinese speakers, vertical spatial metaphors are often used to express temporal information. For example, people often say “last week,” “next month,” “second half of the year,” “ancient era,” etc. Vertical temporal-spatial metaphors are occasionally used in English, they are not as systematic or frequent as in Mandarin where earlier events are called as “Shang” (means up) and later events are called as “Xia” (means down).

Although most studies have supported that time moves from early to late in a top-down direction, some research still found different results that more downward eye movements were detected when recalling the past, and more upward eye movements were detected when imagining the future (Hartmann et al., 2014). Meanwhile, when processing future events, the subject’s eye movement was higher than processing past events (Stocker et al., 2016). Other studies have also pointed out that, whether in the explicit time arrangement task or the implicit spatio-temporal correlation task, the early-up, late-down spatio-temporal mapping was not observed (Yang and Sun, 2018).

Taken together, the direction of vertical MTL is unstable. The instability of the MTL is related to reading and writing habits. Previous studies have found that subjects showed a “right-past, left-future” spatio-temporal mapping after reading the text which was rotated clockwise and counterclockwise (Casasanto and Bottini, 2014; Román et al., 2015). Mandarin people have both left-right and top-down reading and writing habits, as a result, their MTL is multi-directional. Besides, people have a lot of visual experience in their lives. For example, in ancient China, people could know the time by judging the burning height of the incense column (there were fixed intervals on an incense stick, the distance of each interval corresponded to a specific length of time), so the early and late time were characterized in the top-down direction. However, in certain situations, individuals often observe a great number of “rising” scenes, such as balloons flying to the sky and the rocket launching from bottom to top, which means the early time is at the bottom and the late time is on the top. This raises the question of whether vertical representation of time is the result of experience with writing and reading vertically or of visual perception of the physical world. In recent years, the core role of visual experience has been proved by some studies. For example, Bottini et al. (2016) found that sighted and late blind people consistently organize working memory items in space (with early items in the list mapped onto leftward location and later items onto rightward location), however, early blind do not show such consistent spatial mapping, which demonstrated that spatio-temporal mapping was due to visual experience.

According to the embodied cognition theory, time perception is closely related to personal physical activities (Holyoak and Thagard, 1995; Gentner et al., 2002; Barsalou and Wiemer-Hastings, 2005; Gibbs, 2006; Barsalou, 2008). In daily life, everyone will accumulate a variety of perceptual movement experience which is the foundation and key of the connection of time and space (Anelli et al., 2018). Therefore, visual experience might affect people’s concept of mental timeline. To the best of our knowledge, there is no direct experimental evidence to support the role of the visual experience in the vertical dimension of MTL. Meanwhile, we still don’t know whether the vertical MTL is flexible. In this study, life scenarios were used as priming materials of visual experience in order to directly examine the impact of visual experience on the vertical MTL. In experiment 1 and experiment 2, words and events were used as experimental stimuli, and then the subjects were required to complete implicit and explicit time-related judgment task. We assume the direction of vertical MTL will be enhanced in the top-down priming condition and will be weakened or disappear under the bottom-up priming condition.

## Experiment 1

### Method

#### Participants

In this experiment, we estimated the required sample size by using *f* = 0.25 as effect size input in G-Power 3.1.9 (α = 0.05) to detect a medium-sized effect on the main outcomes. The calculation outcome suggested a required sample size of seventy-two in this study. We randomly recruited eighty-five undergraduates and postgraduates, aged from 18 to 24 years old, and they were randomly divided into three groups: twenty-seven subjects were assigned to the “descending group” (15 females, 12 males, mean age = 20.15 ± 1.79), twenty-seven subjects were assigned to the “ascending group” (16 females, 11 males, mean age = 19.74 ± 1.26), and thirty-one people served as the control group (17 females, 14 males, mean age = 20.46 ± 1.30). All participants were right-handed and had a normal or corrected-to-normal vision. No subject quitted during the experiment and everyone was given an appropriate remuneration after the experiment. All participants provided written informed consent before the study, and this study was approved by The Ethics Committee of Sichuan Normal University.

#### Materials

The stimulus was presented on a 17-inch monitor screen with a resolution of 1024 × 768 and a refresh rate of 85 Hz. The image scene was presented in a white rectangle with a side length of 8 cm × 11 cm, which involved two black and white animations of a little man who was ascending and descending a wooden ladder. In each animation, there was a wooden ladder in the vertical direction which had a total height of 9.7 cm and 11 horizontal steps, and each horizontal step was 0.15 cm wide with a 0.7 cm distance between every two steps. The subject’s eyes were about 60 cm away from the screen and kept the same level as the center of the screen during the experiment.

A total of 46 four-character vocabulary related to the “morning and evening” of a day (24 words about the morning and 22 words about the evening) were selected. We asked thirty-eight students to evaluate the familiarity and meaning of these words. According to the scores, a total of 24 words were selected to be used in the formal experiment. There was no significant difference between the morning words (familiarity 4.33 ± 0.19, image degree 3.96 ± 0.23) and the evening words (familiarity 4.38 ± 0.28, image degree 4.00 ± 0.08) [familiarity *t*(22) = –0.54, *p* = 0.60; image degree *t*(22) = –0.41, *p* = 0.69].

#### Procedures

We conducted a mixed design of 2 (time type: early and late) × 2 (response position: above and below) × 3 (priming condition: descending group, ascending group, and control group), with priming condition as a between-subject variable and time type and response position as within-subject variables. The dependent variables are the average response time and accuracy of the vocabulary judgment.

E-prime 2.0 was used to design the program and collect data. In the experiment, the participants in the ascending and descending wooden ladder groups watched the animation first. They could clearly see a person climbing down from the top to bottom or climbing up from the bottom to top, respectively. The animation of descending or ascending the wooden ladder was repeated three times in each group, lasting 3000 ms for each time with an interval of 3000 ms between each time. When the animation finished, the subjects were required to verbally describe it and make sure they had kept the scene in their mind (no less than 1 min). Once subjects make sure that they could keep the scene smoothly, they pressed any key to enter the judgment task. In each trial, a “+” appeared in the middle of the screen for 500 ms, and then a blank screen appeared for 500 ms. When a word appeared, participants were required to distinguish whether the word represent morning or evening by pressing the up or down key within 4000 ms. They pressed the “↑” key if they thought it was earlier than noon and pressed the “↓” key if they thought it was later than noon. There was an interval of 1000 ms between each trial. If the subject did not respond within 4000 ms, the next trial was automatically entered. The control group did not watch the animation and completed the judgment task directly.

Each subject had a certain amount of practice until the correct response rate was over 80 %. Each subject performed a total of 240 trials divided into four blocks. The sequence of keys was balanced among the subjects. Some participants were required to press the “early-up key, late-down key” to respond to the first two blocks, and press the “early-down key, late-up key” to respond to the last two blocks. For other participants, the key sequence was reversed. Participants could take a short break between blocks.

#### Results

After the experiment, all of the subjects reported that they were not clear about the purpose of the experiment. We deleted five subjects’ trials (including three in the descending wooden ladder group and two in the ascending wooden ladder group) because their accuracy rate was below 75 % in a single block. For each participant, the trials with error response and response time within 400 ms and beyond 3500 ms were deleted. The percentage of deleted data was 10.65 %.

The accuracy was analyzed by repeated measures ANOVA. The main effect of time type was significant, *F* (1,77) = 11.53, *p* = 0.001, η_*p*_^2^ = 0.13, and the correct rate of response to late words was significantly higher than that to early words. The main effect of priming condition was not significant, *F*(2,77) = 1.91, *p* = 0.15, η_*p*_^2^ = 0.05. The interaction effect between time type and response position was significant, *F*(1,75) = 6.49, *p* = 0.013, η_*p*_^2^ = 0.08. Importantly, the interaction effect among time type, response position, and priming condition was approximately significant, *F*(2,77) = 3.08, *p* = 0.052, η_*p*_^2^ = 0.07. Simple effect analysis showed that in the ascending group, the accuracy of pressing the “up” key was significantly higher than that of pressing the “down” key when responding to early words (0.96 ± 0.01 vs. 0.93 ± 0.01), *F*(1,77) = 6.89, *p* = 0.01, η_*p*_^2^ = 0.08, and the accuracy of pressing the “down” key was significantly higher than that of pressing the “up” key when responding to late words, (0.97 ± 0.01 vs. 0.94 ± 0.01), *F*(1,77) = 6.63, *p* = 0.012, η_*p*_^2^ = 0.08. In the descending group and the control group, the interaction was not significant (*p* > 0.05).

**FIGURE 1:**
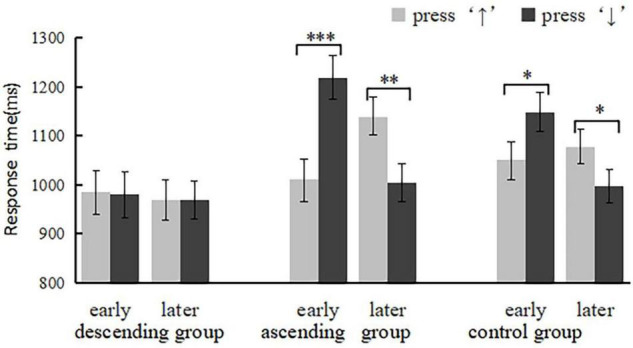
Results from Experiment 1: mean response time as a function of priming condition (ascending group, descending group and the control group) and time type (early and later) in pressing “↑”(gray bars) or “↓”(black bars) conditions. The Error bars represent the standard errors for each condition. *p < 0.05; **p < 0.01; ***p < 0.001.

The reaction times were analyzed by repeated measures ANOVA. The main effect of time type was significant, *F*(1,77) = 13.02, *p* = 0.001, η_*p*_^2^ = 0.15, that the responses to late words were significantly faster than those to early words, (1025.44 ± 19.12 ms vs. 1065.94 ± 21.28 ms). The main effect of priming condition was significant, *F*(2,77) = 3.20, *p* = 0.046, η_*p*_^2^ = 0.08. The *post hoc* analysis showed that the descending group responded significantly faster than the ascending group (975.35 ± 35.26 ms vs.1093.33 ± 34.55 ms) and than the control group (975.35 ± 35.26 ms vs. 1068.39 ± 31.02 ms). The interaction effect between time type and response position was significant, *F*(1,77) = 14.34, *p* < 0.001, η_*p*_^2^ = 0.16. The interaction among word type, response position, and task type was significant, *F*(2,77) = 4.69, *p* = 0.012, η_*p*_^2^ = 0.11. Simple effect analysis to the control group found that subjects responded faster when pressing the “up” key to early words [*F*(1,77) = 6.30, *p* = 0.014, η_*p*_^2^ = 0.08] and pressing the “down” key to late words [*F*(1,77) = 4.53, *p* = 0.036, η_*p*_^2^ = 0.06], which indicated that the direction of the MTL was from top to down. In the ascending group, subjects responded faster when pressing the “up” key to early words [*F*(1,77) = 23.02, *p* < 0.001, η_*p*_^2^ = 0.23] and pressing the “down” key to late words [*F*(1,77) = 10.66, *p* = 0.002, η_*p*_^2^ = 0.12], which showed that the ascending group did not show an expected opposite MTL. While in the descending group, the interaction effect between word type and response position was not significant, demonstrating the up-down mental timeline disappeared (see Figure 1).

Experiment 1 showed that participants’ response to late stimuli was significantly faster than that to early stimuli. The time range in Experiment 1 was limited to one day. According to the general life rhythm, in the morning, people are usually busy at work and school, and in the evening and night, they have more leisure time to observe and experience the surrounding environment. Therefore, people respond more quickly to late words as a result of having more experience of sunset and night.

Reaction time revealed that the control group showed an “early-up, late-down” spatio-temporal mapping, which was in line with our expectation. However, contrary to the expectation, when we asked subjects to watch up-down or down-up scenes, we did not observe an opposite MTL. But in the study of Chen (2018), after joining the virtual sensation movement of up and down of the elevator, the top-down MTL did not appear. The different results may be due to “safety” reasons. Ascending wood ladder situation may cause subjects’ safety anxiety, which made them want to be closer to the land, inhibiting the emergence of spatio-temporal association. While in the descending wooden ladder situation, the task itself was more in line with the subjects’ safety needs and did not consume too many cognitive resources, which lead to faster response. In experiment 1, we used implied time information that did not directly express time, which might cause the expected results did not to appear. In Experiment 2, we used materials that represent time directly to further investigate the role of visual experience under implicit and explicit conditions.

## Experiment 2

### Method

#### Participants

In this experiment, we estimated the required sample size by using *f* = 0.25 as effect size input in G-Power 3.1.9 (α = 0.05) to detect a medium-sized effect on the main outcomes. The calculation outcome suggested a required sample size of seventy-two for the experiment. A total of eighty-seven undergraduates and postgraduates were randomly divided into three groups. The first group contained a total of thirty participants (17 females and 13 males, mean age = 19.17 ± 1.23). The second and control group were composed of twenty-eight participants (17 females and 11 males mean age = 19.79 ± 1.91) and twenty-nine participants (20 females and 9 males, mean age = 19.62 ± 1.76). All participants were right-handed and had a normal or corrected-to-normal vision. No subject quitted during the experiment and everyone was given an appropriate remuneration after the experiment. All participants provided written informed consent before the study, and this study was approved by The Ethics Committee of Sichuan Normal University.

#### Materials

**FIGURE 2:**
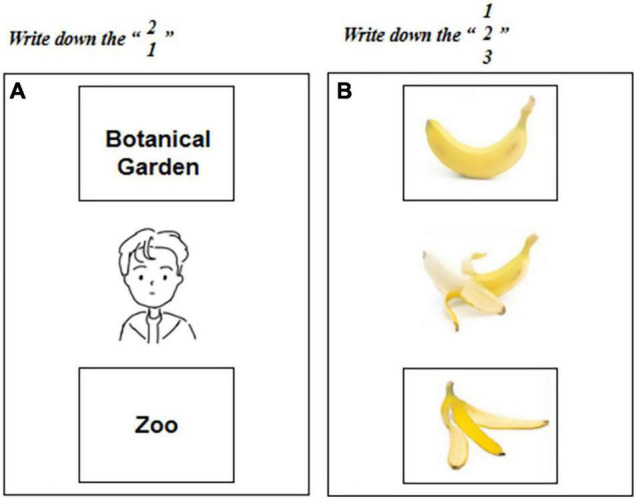
Order diagram of event occurrence **(A,B)**. There is a character in the middle of the first board with empty squares above and below the character **(A)**. The mid-term picture of the occurrence of the event is presented in the middle of the second board with two empty squares above and below the mid-term picture **(B)**.

(1) Video: There were a total of 10 videos with 5 descending videos (e.g., apple falling) and 5 ascending videos (e.g., balloon flying away). The videos were presented on a 17-inch monitor screen with a 1024 × 768 screen resolution and a refresh rate of 85 Hz.
(2) Cards: There were 15 sets of cards in sequence of events and each set of cards included 3 cards (12 cm × 12 cm) which represented the early, middle and late states of the event (see Supplementary Figure 1).
(3) Filling board: There was a character in the middle of the first board with empty squares above and below the character (see Figure 2A). The mid-term picture of the occurrence of the event was presented in the middle of the second board with two empty squares above and below the mid-term picture (see Figure 2B).
(4) Voice material: The recording software (version 2.2.0 of the mobile APP “Recording Expert”) was used to record. The material itself did not contain any direction, but there were 30 Chinese items such as “Xiao Ming visited the zoo yesterday and will visit the botanical garden tomorrow.”

#### Procedures

A two-factor mixed design of 3 (priming direction: downward, upward and none priming group) × 2 (task type: implicit and explicit) was adopted, in which priming direction was taken as the between-subject variable and task type as within-subject variable.

Each participant first watched 5 downward or upward videos in sequence, and tried to use one sentence to briefly describe what they had just seen after each video. And then they completed the time chart task and card sorting task. In the time chart task, the subjects first listened to a sentence, such as “Xiao Ming visited the zoo yesterday and will visit the botanical garden tomorrow” which was repeated twice, then they orally repeated the main content of the sentence to make sure they had heard it clearly. Finally the subjects wrote the words “zoo” and “botanical garden” into the box within 15s, and those positions written by the participants were recorded by the examiner (see Supplementary Table 2 record 1). The subjects were not allowed to modify their answers once they finished the task. Subsequently, each subject received a set of cards which represent early and late states of the event, and placed it into the upper and lower boxes in the second board according to the sequence of events. The examiner recorded the position of the cards placed by the participant (see Supplementary Tables 1, 2 record 2). The specific recording method is as follows:

Record 1: Audio material “Xiao Ming visited the zoo yesterday, and will visit the botanical garden tomorrow”:

The zoo was on the top and the botanical garden was on the bottom (the early words associate with up and the late words associate with down) were marked as “
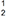
”;

The botanical garden was on the top and the zoo was on the bottom (the early words associate with down and the late words associate with up) were marked as “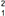.”

Record 2: Top-down was marked as 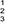; bottom-up was marked as 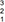.

#### Data Analysis

According to records 1 and 2, respectively, the percentage of participants who wrote the names of the early things or put the early pictures in the “up” position was calculated and the ratio of the “bottom-up” direction = 1-the ratio of the “top-down” direction.

#### Results

**FIGURE 3:**
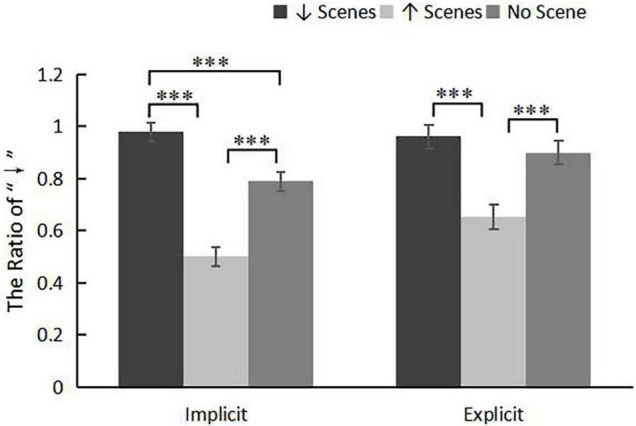
Results from Experiment 2: mean ration of “↓” as a function of task type in downward priming (black bars), upward priming (light gray bars) and none priming conditions (dark gray bars). The Error bars represent the standard errors for each condition. ***p < 0.001.

We took the percentage of top-down arrangement as the dependent variable, and conduct ANOVA of repeated measure with 2 (task type: implicit and explicit) × 3 (priming direction: downward, upward, and none priming condition). The main effect of task type was significant, *F*(1, 84) = 8.24, *p* = 0.005, η_*p*_^2^ = 0.09. The implicit group had a lower average selection ratio than the explicit group (0.76 ± 0.02 vs. 0.84 ± 0.03). The main effect of priming direction was significant, *F*(2, 84) = 35.87, *p* < 0.001, η_*p*_^2^ = 0.46. The interaction effect was significant, *F*(2, 84) = 3.21, *p* = 0.045, η_*p*_^2^ = 0.07 (see Figure 3). Simple effect analysis showed that in the implicit task, the selection ratio of downward priming group was higher than that of upward priming group (0.98 ± 0.04 vs. 0.50 ± 0.04), *p* < 0.001; the selection ratio of downward priming group was higher than that of the control group (0.98 ± 0.04 vs. 0.79 ± 0.04), *p* < 0.001; the selection ratio of the control group was higher than that of upward priming group(0.79 ± 0.04 vs. 0.50 ± 0.04), *p* < 0.001. In the explicit task, the results were similar. The selection ratio of downward priming group was higher than that of the upward priming group (0.96 ± 0.05 vs. 0.65 ± 0.05), *p* < 0.001; the selection ratio of the control group was higher than that of the upward priming group (0.90 ± 0.05 vs. 0.65 ± 0.05), *p* < 0.001. But the difference between the downward priming group and the control group was not significant (0.96 ± 0.05 vs. 0.90 ± 0.05, *p* > 0.05).

In the implicit and explicit tasks, the ratio of participants’ choice of top-down and bottom-up directions was tested by single sample *T*-test, and the compared value was 0.5. The results showed that in the downward priming group, the participants tended to choose the top-down direction in both implicit (*t* = 60.69, *df* = 29, *p* < 0.001, *d* = 22.54) and explicit tasks (*t* = 21.31, *df* = 29, *p* < 0.001, *d* = 7.91). In the upward priming group, the participants in the implicit task had no orientation tendency, *t* = 0.001, *df* = 27, *p* > 0.05; while in the explicit task, the participants tended to choose the top-down direction, *t* = 2.07, *d f* = 27, *p* = 0.049, *d* = 0.79. In the control group, participants tended to choose the top-down direction in both implicit (*t* = 9.29, *df* = 28, *p* < 0.001, *d* = 3.51) and explicit tasks (*t* = 13.66, *df* = 28, *p* < 0.001, *d* = 5.16).

In Experiment 2, compared with the control condition, the downward priming enhanced the top-down MTL at the implicit and explicit level, and the upward priming weakened the top-down MTL at the implicit level. It showed that when the experimental stimulus was directly related to time, the vertical MTL was affected by the experience of perceptual movement. Existing studies have also found that after reading sentences containing top-down or forward-to-backward metaphors, subjects were more inclined to construct time representation that adopt the corresponding trend (Lai and Boroditsky, 2013; Hendricks and Boroditsky, 2017). But one study found that visual experience had no effect on the vertical MTL (Chen, 2018). The reason may be that the subject’s own movement was opposed to the motion scene they watched. In our study, participants’ visual experience was separated from their own motion, and the results found that visual experience in different directions had a significant impact on the MTL.

## General Discussion

In alphabetic languages such as English, mental timeline are exclusively in a horizontal manner, whereas Mandarin can be either horizontally or vertically. It was suggested that Mandarin speakers’ vertical conceptualization of time may be the result of the experience of writing and reading vertically (Chan and Bergen, 2005; Bergen and Chan, 2012; Chen and O’Seaghdha, 2013). Fuhrman et al. (2011) and Miles et al. (2011) also reported a strong vertical pattern in Mandarin speakers with more experience in writing and reading vertical texts, suggesting that vertical spatio-temporal metaphors used in the language should have a stronger influence on the conceptualization of time.

Researchers have found the spatio-temporal mapping of “past-up, future-down” by using different reaction methods such as manual buttons pressing, eye movement position, free placement task in three-dimensional space and so on (Boroditsky et al., 2011; Gu et al., 2017). However, these studies have thus far focused only on vertical representations of time, which has not much exploration about whether the mental timeline is stable in the vertical direction and the role of visual experience other than writing and reading. The MTL in the vertical direction is very unstable, as this study shows that when task stimuli directly express time information, perceptual experience can significantly change the MTL.

## The Role of Visual Experience

Visual experience is an important factor affecting the MTL. In Experiment 2, subjects’ visual experience of watching other things moving was separated from their own sensory movement, and the results found that visual experience in different directions had a significant impact on the vertical MTL. From the perspective of embodied theory, perceptual experience plays an important role in the formation of MTL. Since people recognize the surrounding environment through observing and playing in their early stage, they accumulate a large number of spatio-temporal related scenes in vision and motor perception, which form the relationship between the concept of time and the concept of space. Multiple situational stimuli make this relationship repeatedly strengthened and eventually form the spatial representation of time.

However, the priming effect of perceptual experience is related to the task. When the time task is not relevant, some studies have not found the automatic activation of the MTL (Ulrich and Maienborn, 2010; Ulrich et al., 2012; Maienborn et al., 2015; von Sobbe et al., 2019). The activation of the MTL depends on the level of temporal complexity (Scheifele et al., 2018). When time has high complexity and has nothing to do with the task, the spatio-temporal mapping might disappear. In Experiment 1, the stimuli were words with high complexity which contain implicit time information, thus subjects may concentrate on processing and understanding the time information of the material without using spatial information. At this time, the degree of spatial activation was low, so the results showed that ascending and descending the wooden ladder had no impact on the direction of the vertical MTL.

## The Difference Between Implicit and Explicit Spatio-Temporal Mapping

This study also investigated the implicit and explicit spatio-temporal mapping. The results of the control group showed that the subjects had a significant advantage in the top-down direction regardless of the implicit or explicit levels, supporting the previous view that subjects had a top-down mental timeline (Boroditsky, 2001; Boroditsky et al., 2011; Fuhrman et al., 2011; Miles et al., 2011). Existing studies have found the implicit spatio-temporal mapping mainly in the front and back dimensions. de la Fuente et al. (2014) used the method of cognitive training to randomly assign Spanish speakers to the “past focus” and “future focus” groups, and found that in the time chart task, the frequency of answering “the past is in front and the future behind” increased when participants received past focus training, and the frequency of answering “the future is in front and the past behind” increased when participants received future focus training. Although the two groups of subjects had the same linguistic and cultural background, their implicit spatio-temporal mapping directions were also different due to the different time focus of their attention. Other researchers also found that subjects did not show obvious preferences in the front and back dimensions by carrying out the time chart task; while the implicit spatio-temporal mapping of Qiang subjects was “past-before, future-after” (Li and Cao, 2018). In this study, when the MTL was activated by upward perceptual experience, the original “early-up, late-down” spatio-temporal mapping was weakened at both implicit and explicit levels, and the weakening effect was even more significant at the implicit level. Together with previous studies, this study shows that human implicit space-time mapping has greater flexibility and plasticity which could be changed with people’s perceptual experience.

## Conclusion

In this paper, we provided an investigation of the influence of visual experience on the vertical MTL. The results showed that the MTL on the vertical axis was not activated automatically when the temporal information was not prominent. And the MTL on the vertical axis could be changed in both explicit and implicit tasks, and the changes were more obvious under implicit conditions. To date, none of studies have considered visual experience as a variable when investigating vertical mental timeline. It is therefore expected that our research will attract more attention of researchers to investigate the vertical MTL.

## Data Availability Statement

The original contributions presented in the study are included in the article/Supplementary Material, further inquiries can be directed to the corresponding author.

## Ethics Statement

The studies involving human participants were reviewed and approved by the Ethics Committee of Sichuan Normal University and all the procedures involved were in line with the sixth revision of the Helsinki Declaration. The patients/participants provided their written informed consent to participate in this study. Written informed consent was obtained from the individual(s) for the publication of any potentially identifiable images or data included in this article.

## Author Contributions

CB contributed to conception. JH and CB designed the experiment and wrote the manuscript. JH carried out the experiment and analyzed the data. HJ and JM modified the manuscript and refined the language. All authors contributed to manuscript revision, read, and approved the submitted version.

## Conflict of Interest

The authors declare that the research was conducted in the absence of any commercial or financial relationships that could be construed as a potential conflict of interest.

## Publisher’s Note

All claims expressed in this article are solely those of the authors and do not necessarily represent those of their affiliated organizations, or those of the publisher, the editors and the reviewers. Any product that may be evaluated in this article, or claim that may be made by its manufacturer, is not guaranteed or endorsed by the publisher.

## Supplementary Material

Supplementary Figure 1 Order diagram of event occurrence. Each set of cards included 3 cards (12 cm × 12 cm) which represented the early, middle and late states of the event.

Supplementary Table 1 Results Record Table of Card sorting task (Section). First record the judgment of the two small cards as “start” or “last”, correctly call “√” under “R” and “√” under “W”; then call “√” in the corresponding position in the table in the direction represented by the display itself.

Supplementary Table 2 Record table of implicit and explicit task results (section). According to the placement results of the subject, play “√” in the corresponding position in the table according to the direction of the placement representative.
