# Supplementary figures and images for: The Variability of Mental Timeline in Vertical Dimension
Source: Front Psychol. 2021 Dec 31;12:782975. doi: 10.3389/fpsyg.2021.782975 (PMC8759226; doi:10.3389/fpsyg.2021.782975)

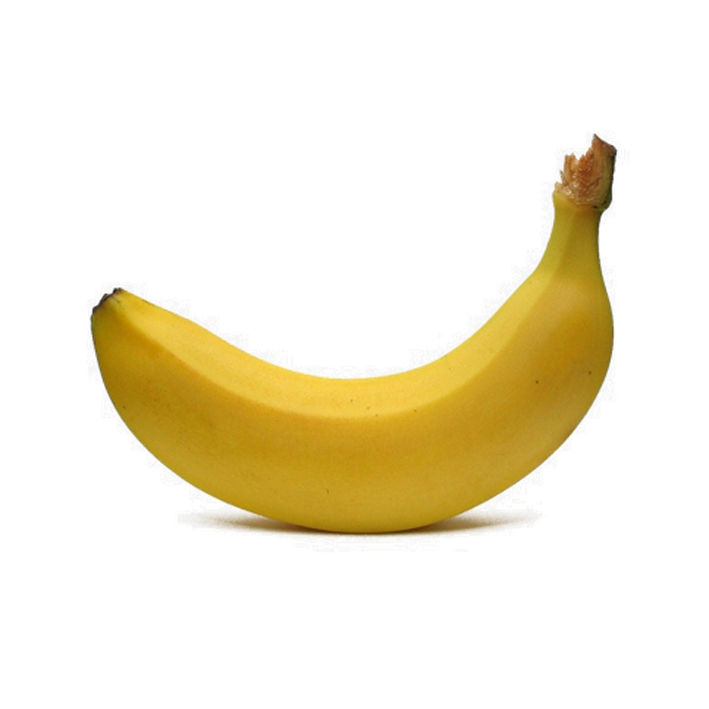

Supplement: Supplementary Figure 1 — Order diagram of event occurrence. Each set of cards included 3 cards (12 cm × 12 cm) which represented the early, middle and late states of the event. [file Data_Sheet_1.zip › Exp2new-Cards in sequence of events/═╝╞1⁄41.png]

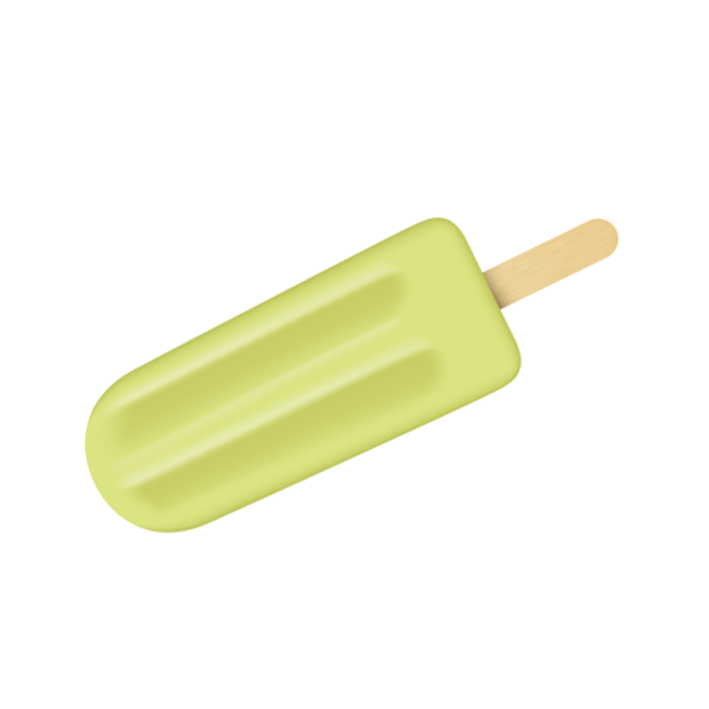

Supplement: Supplementary Figure 1 — Order diagram of event occurrence. Each set of cards included 3 cards (12 cm × 12 cm) which represented the early, middle and late states of the event. [file Data_Sheet_1.zip › Exp2new-Cards in sequence of events/═╝╞1⁄410.png]

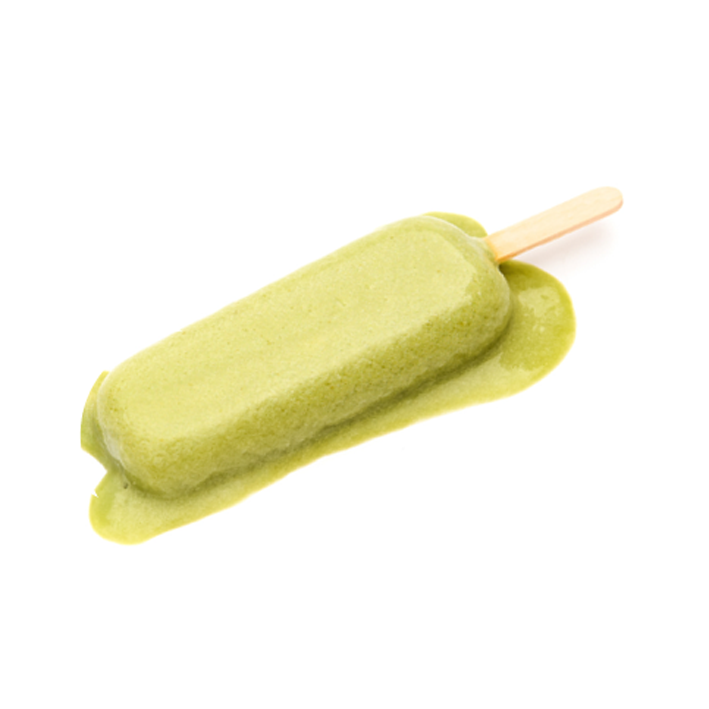

Supplement: Supplementary Figure 1 — Order diagram of event occurrence. Each set of cards included 3 cards (12 cm × 12 cm) which represented the early, middle and late states of the event. [file Data_Sheet_1.zip › Exp2new-Cards in sequence of events/═╝╞1⁄411.png]

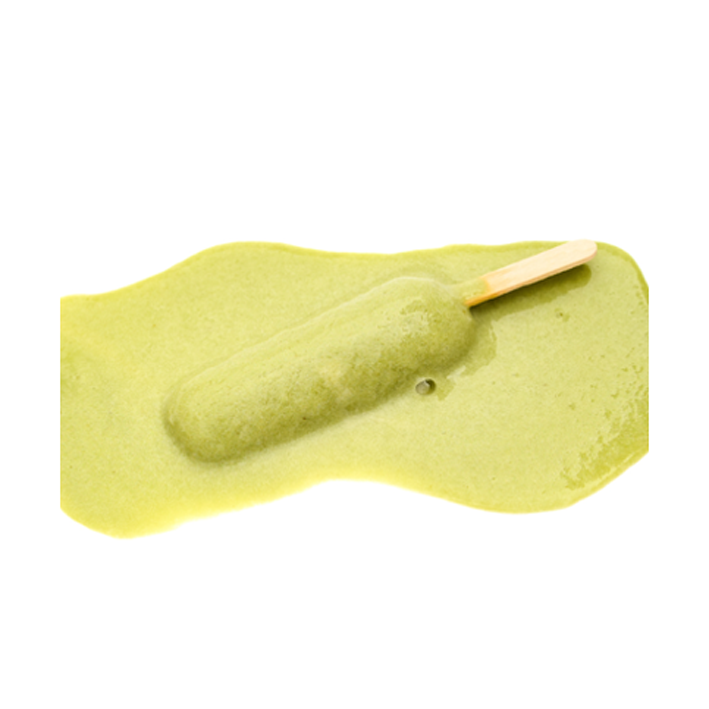

Supplement: Supplementary Figure 1 — Order diagram of event occurrence. Each set of cards included 3 cards (12 cm × 12 cm) which represented the early, middle and late states of the event. [file Data_Sheet_1.zip › Exp2new-Cards in sequence of events/═╝╞1⁄412.png]

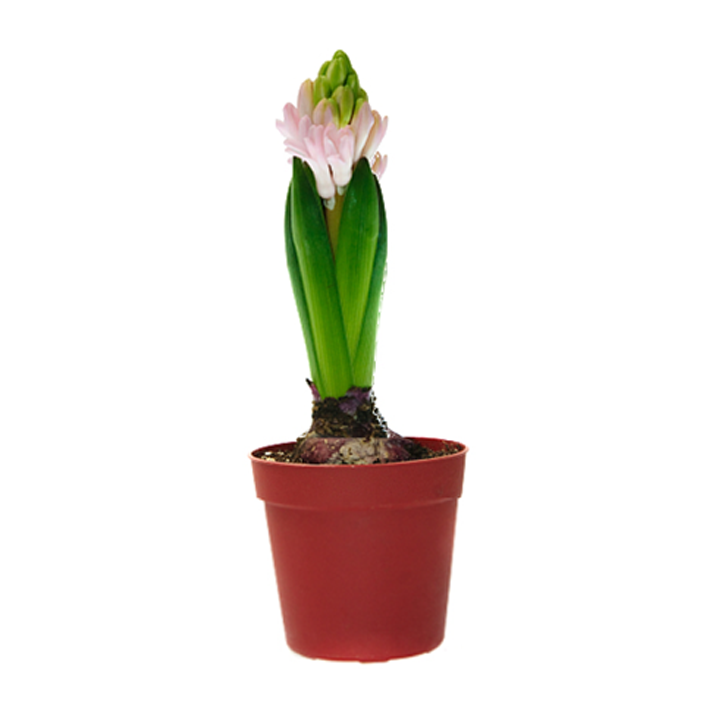

Supplement: Supplementary Figure 1 — Order diagram of event occurrence. Each set of cards included 3 cards (12 cm × 12 cm) which represented the early, middle and late states of the event. [file Data_Sheet_1.zip › Exp2new-Cards in sequence of events/═╝╞1⁄413.png]

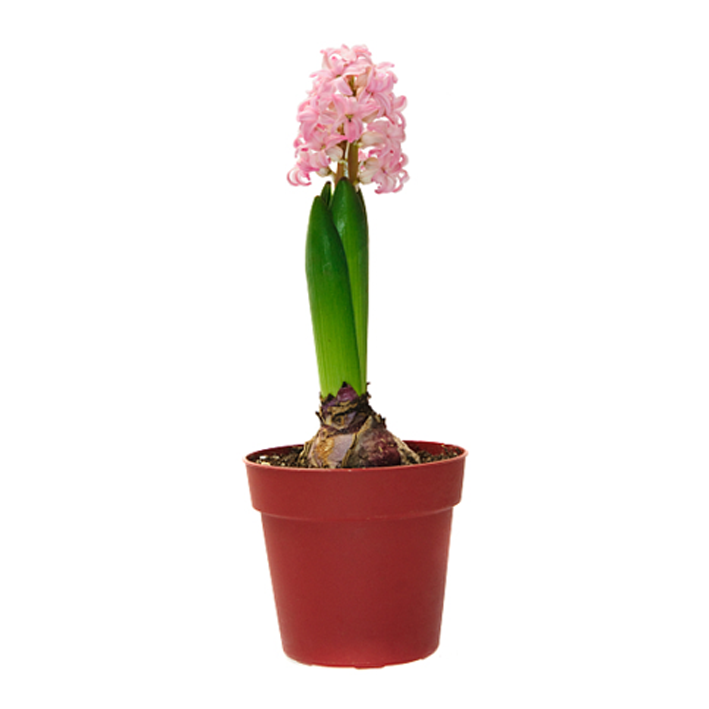

Supplement: Supplementary Figure 1 — Order diagram of event occurrence. Each set of cards included 3 cards (12 cm × 12 cm) which represented the early, middle and late states of the event. [file Data_Sheet_1.zip › Exp2new-Cards in sequence of events/═╝╞1⁄414.png]

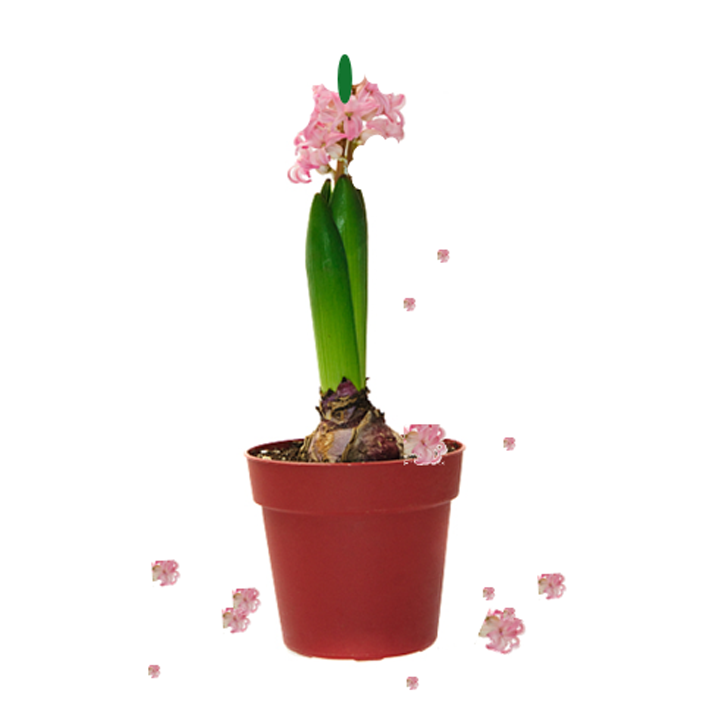

Supplement: Supplementary Figure 1 — Order diagram of event occurrence. Each set of cards included 3 cards (12 cm × 12 cm) which represented the early, middle and late states of the event. [file Data_Sheet_1.zip › Exp2new-Cards in sequence of events/═╝╞1⁄415.png]

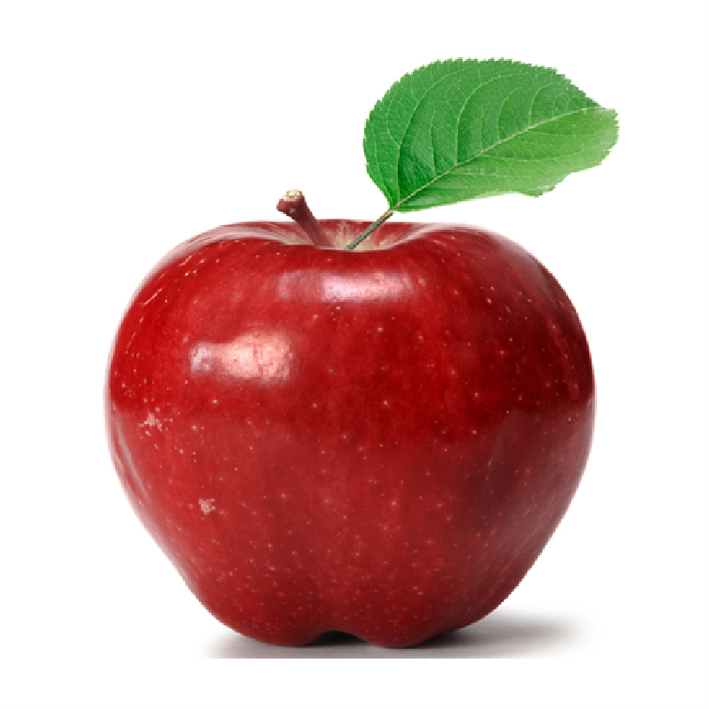

Supplement: Supplementary Figure 1 — Order diagram of event occurrence. Each set of cards included 3 cards (12 cm × 12 cm) which represented the early, middle and late states of the event. [file Data_Sheet_1.zip › Exp2new-Cards in sequence of events/═╝╞1⁄416.png]

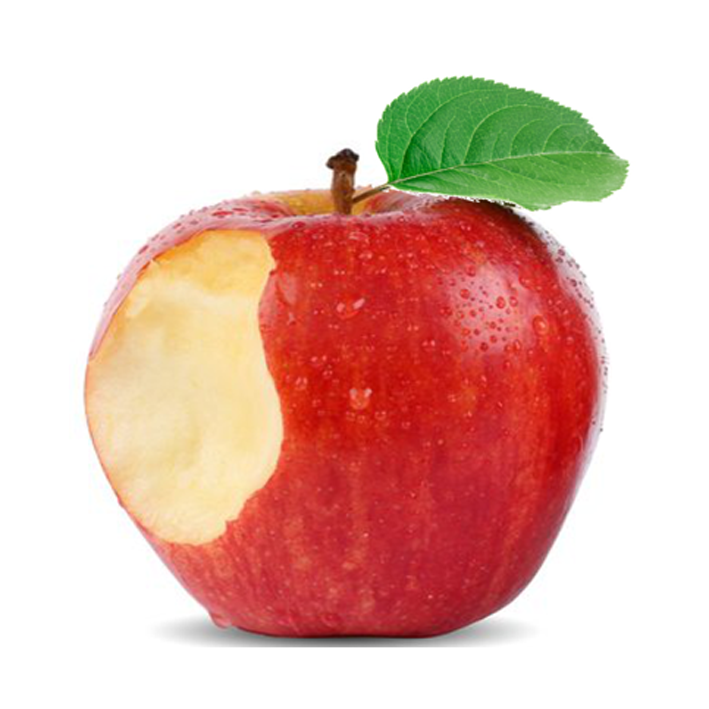

Supplement: Supplementary Figure 1 — Order diagram of event occurrence. Each set of cards included 3 cards (12 cm × 12 cm) which represented the early, middle and late states of the event. [file Data_Sheet_1.zip › Exp2new-Cards in sequence of events/═╝╞1⁄417.png]

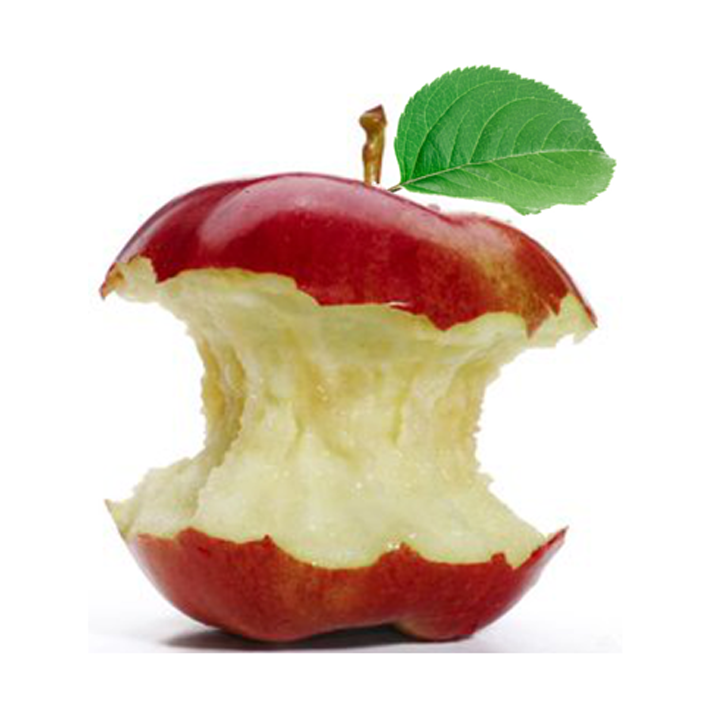

Supplement: Supplementary Figure 1 — Order diagram of event occurrence. Each set of cards included 3 cards (12 cm × 12 cm) which represented the early, middle and late states of the event. [file Data_Sheet_1.zip › Exp2new-Cards in sequence of events/═╝╞1⁄418.png]

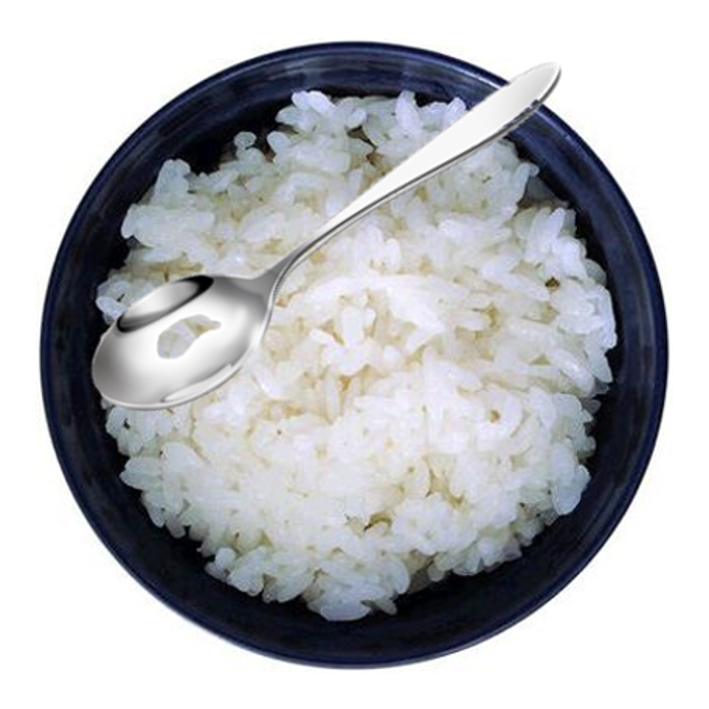

Supplement: Supplementary Figure 1 — Order diagram of event occurrence. Each set of cards included 3 cards (12 cm × 12 cm) which represented the early, middle and late states of the event. [file Data_Sheet_1.zip › Exp2new-Cards in sequence of events/═╝╞1⁄419.png]

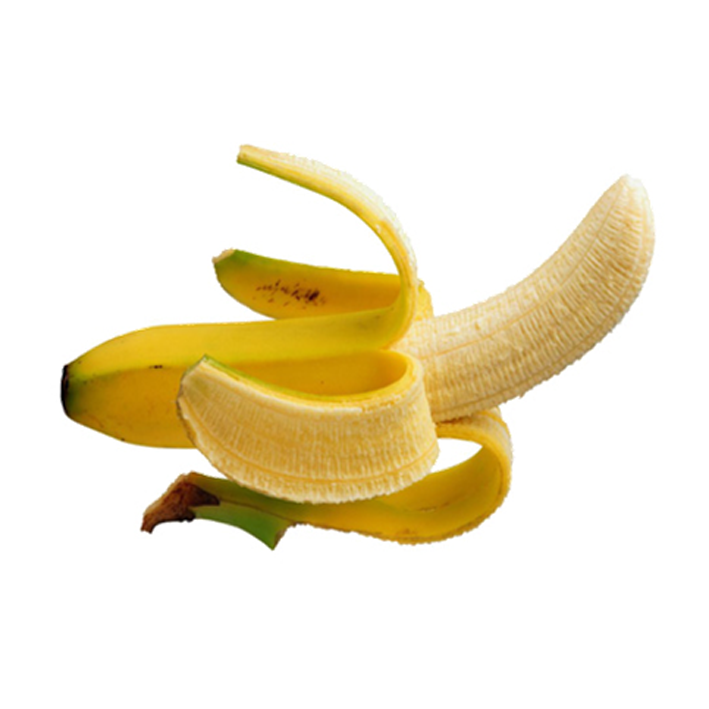

Supplement: Supplementary Figure 1 — Order diagram of event occurrence. Each set of cards included 3 cards (12 cm × 12 cm) which represented the early, middle and late states of the event. [file Data_Sheet_1.zip › Exp2new-Cards in sequence of events/═╝╞1⁄42.png]

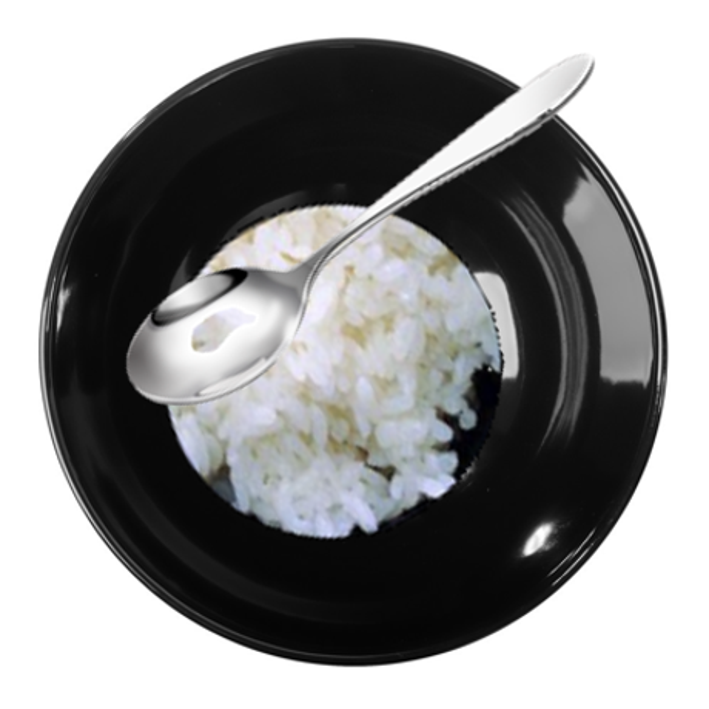

Supplement: Supplementary Figure 1 — Order diagram of event occurrence. Each set of cards included 3 cards (12 cm × 12 cm) which represented the early, middle and late states of the event. [file Data_Sheet_1.zip › Exp2new-Cards in sequence of events/═╝╞1⁄420.png]

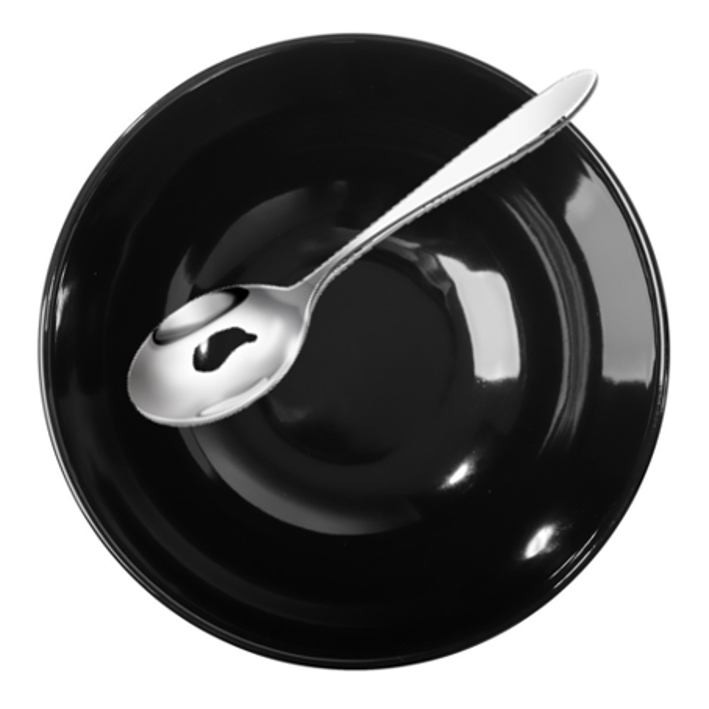

Supplement: Supplementary Figure 1 — Order diagram of event occurrence. Each set of cards included 3 cards (12 cm × 12 cm) which represented the early, middle and late states of the event. [file Data_Sheet_1.zip › Exp2new-Cards in sequence of events/═╝╞1⁄421.png]

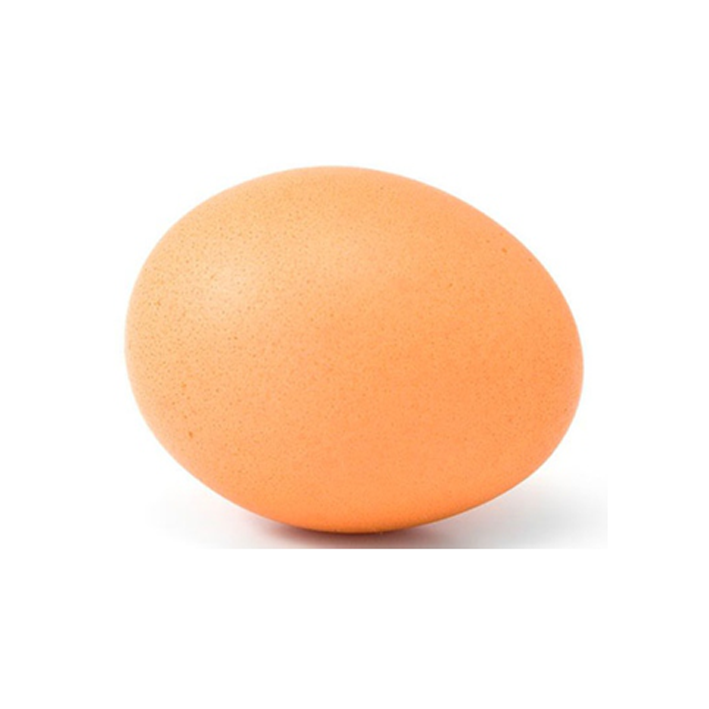

Supplement: Supplementary Figure 1 — Order diagram of event occurrence. Each set of cards included 3 cards (12 cm × 12 cm) which represented the early, middle and late states of the event. [file Data_Sheet_1.zip › Exp2new-Cards in sequence of events/═╝╞1⁄422.png]

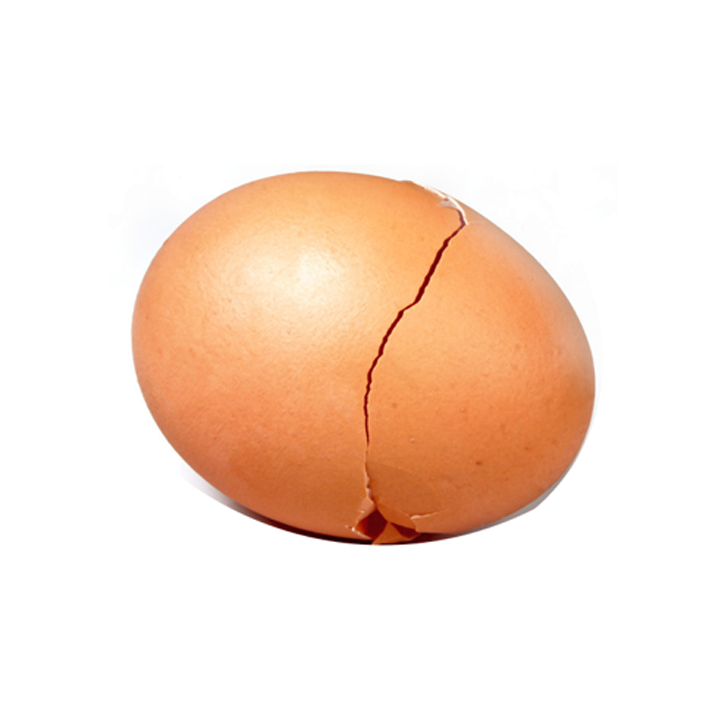

Supplement: Supplementary Figure 1 — Order diagram of event occurrence. Each set of cards included 3 cards (12 cm × 12 cm) which represented the early, middle and late states of the event. [file Data_Sheet_1.zip › Exp2new-Cards in sequence of events/═╝╞1⁄423.png]

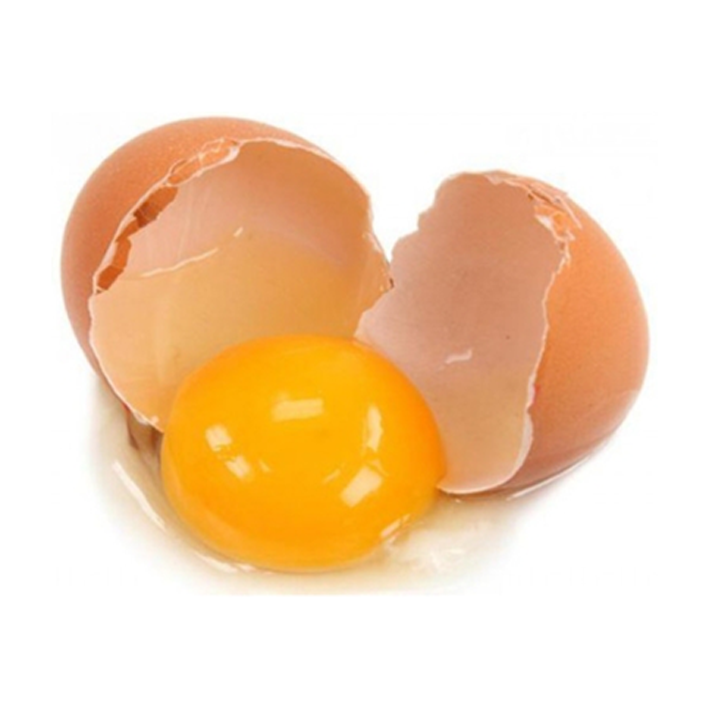

Supplement: Supplementary Figure 1 — Order diagram of event occurrence. Each set of cards included 3 cards (12 cm × 12 cm) which represented the early, middle and late states of the event. [file Data_Sheet_1.zip › Exp2new-Cards in sequence of events/═╝╞1⁄424.png]

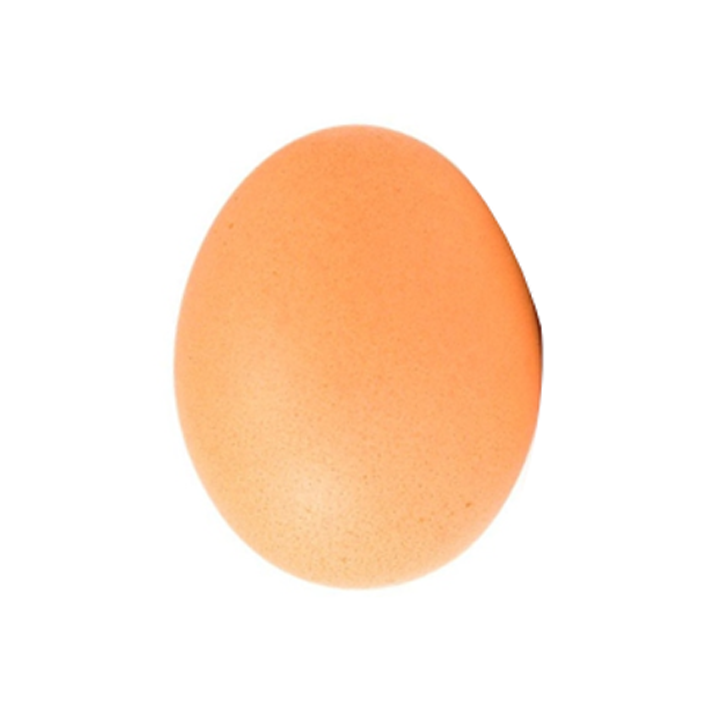

Supplement: Supplementary Figure 1 — Order diagram of event occurrence. Each set of cards included 3 cards (12 cm × 12 cm) which represented the early, middle and late states of the event. [file Data_Sheet_1.zip › Exp2new-Cards in sequence of events/═╝╞1⁄425.png]

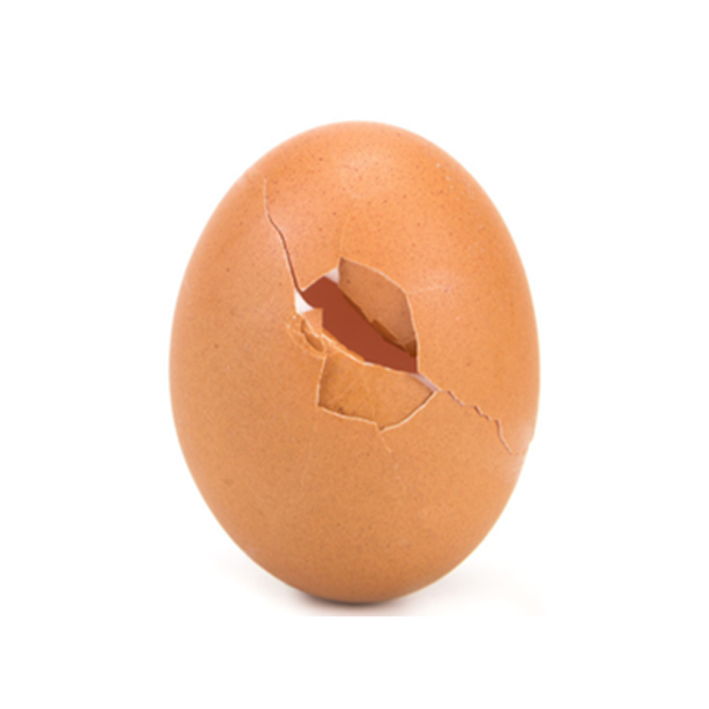

Supplement: Supplementary Figure 1 — Order diagram of event occurrence. Each set of cards included 3 cards (12 cm × 12 cm) which represented the early, middle and late states of the event. [file Data_Sheet_1.zip › Exp2new-Cards in sequence of events/═╝╞1⁄426.png]

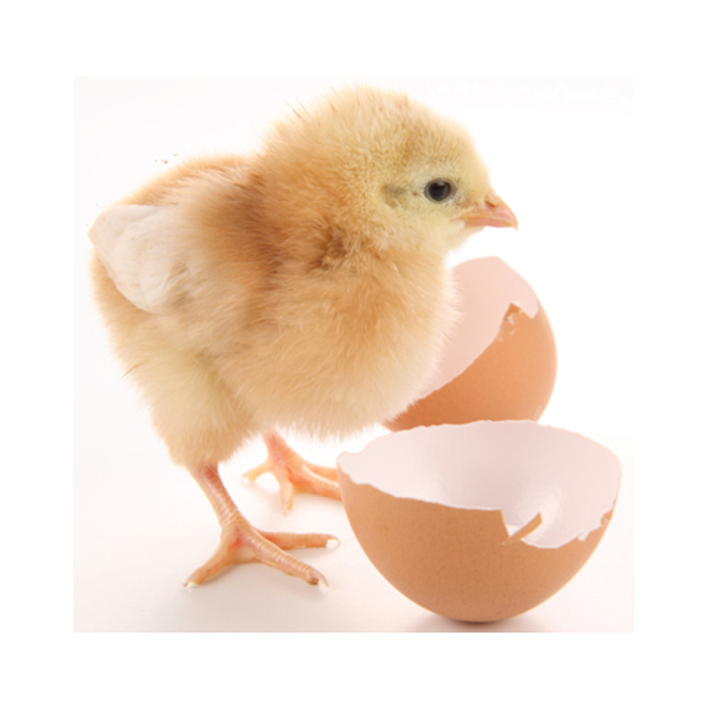

Supplement: Supplementary Figure 1 — Order diagram of event occurrence. Each set of cards included 3 cards (12 cm × 12 cm) which represented the early, middle and late states of the event. [file Data_Sheet_1.zip › Exp2new-Cards in sequence of events/═╝╞1⁄427.png]

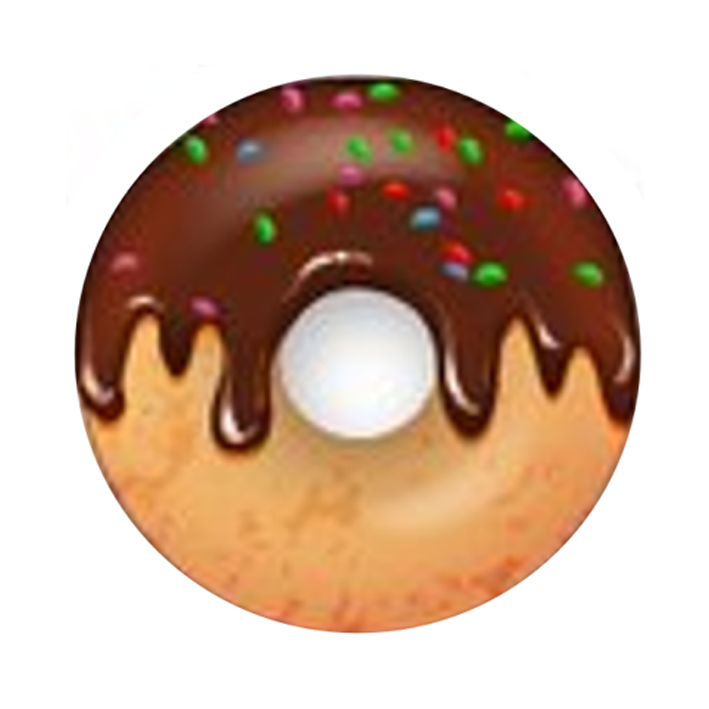

Supplement: Supplementary Figure 1 — Order diagram of event occurrence. Each set of cards included 3 cards (12 cm × 12 cm) which represented the early, middle and late states of the event. [file Data_Sheet_1.zip › Exp2new-Cards in sequence of events/═╝╞1⁄428.png]

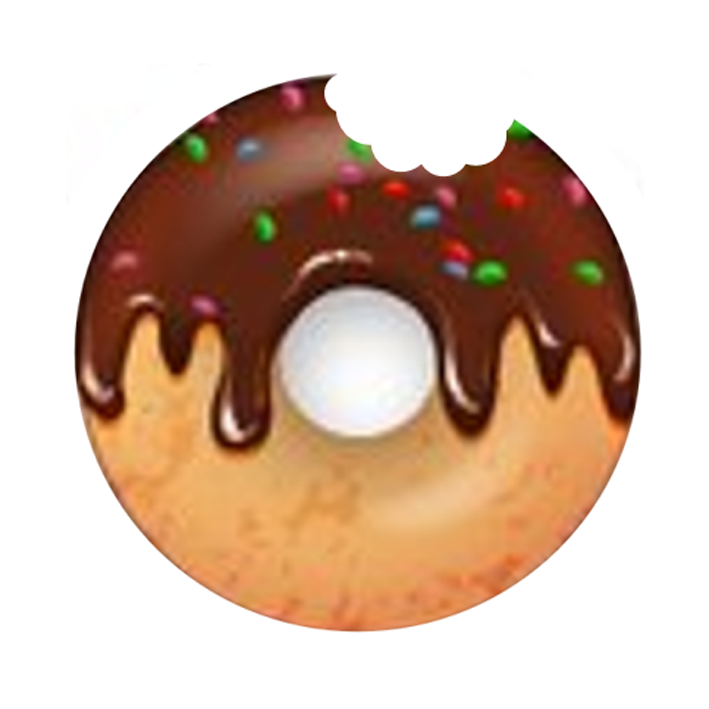

Supplement: Supplementary Figure 1 — Order diagram of event occurrence. Each set of cards included 3 cards (12 cm × 12 cm) which represented the early, middle and late states of the event. [file Data_Sheet_1.zip › Exp2new-Cards in sequence of events/═╝╞1⁄429.png]

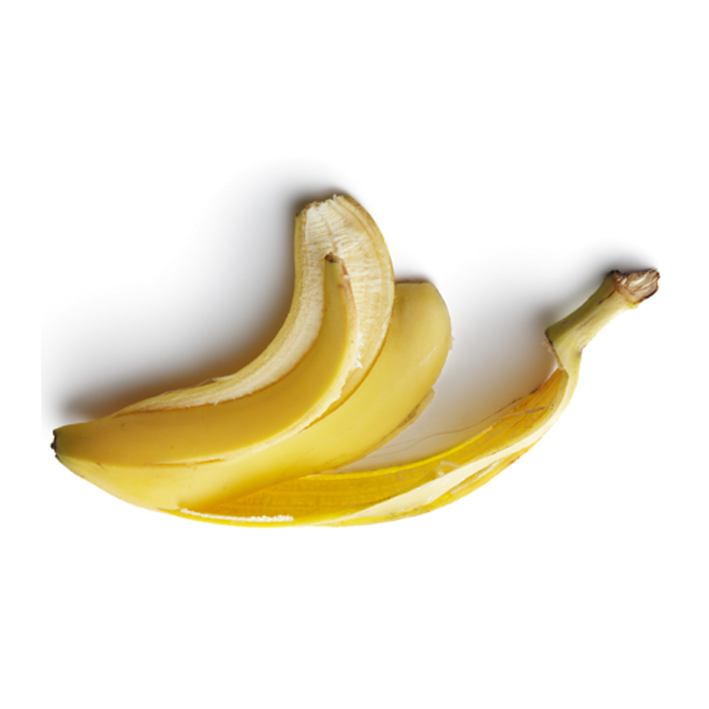

Supplement: Supplementary Figure 1 — Order diagram of event occurrence. Each set of cards included 3 cards (12 cm × 12 cm) which represented the early, middle and late states of the event. [file Data_Sheet_1.zip › Exp2new-Cards in sequence of events/═╝╞1⁄43.png]

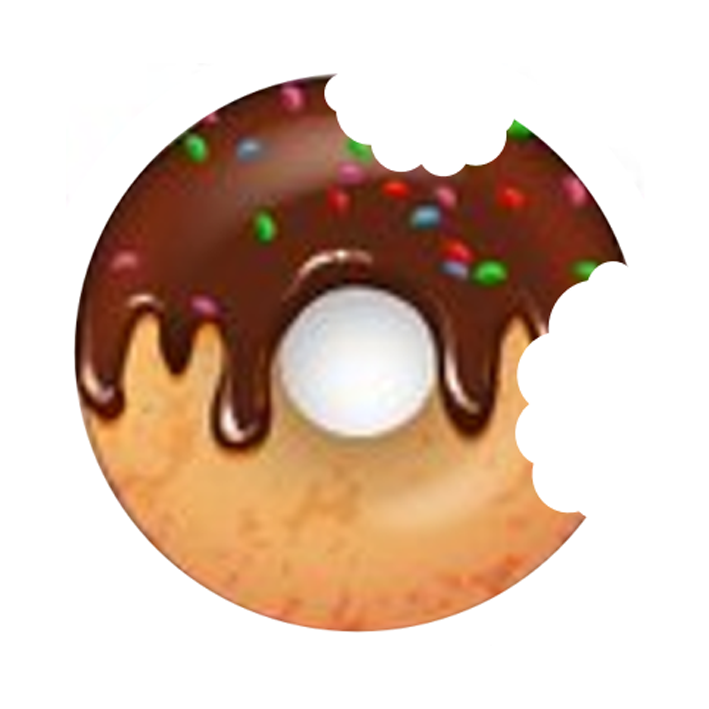

Supplement: Supplementary Figure 1 — Order diagram of event occurrence. Each set of cards included 3 cards (12 cm × 12 cm) which represented the early, middle and late states of the event. [file Data_Sheet_1.zip › Exp2new-Cards in sequence of events/═╝╞1⁄430.png]

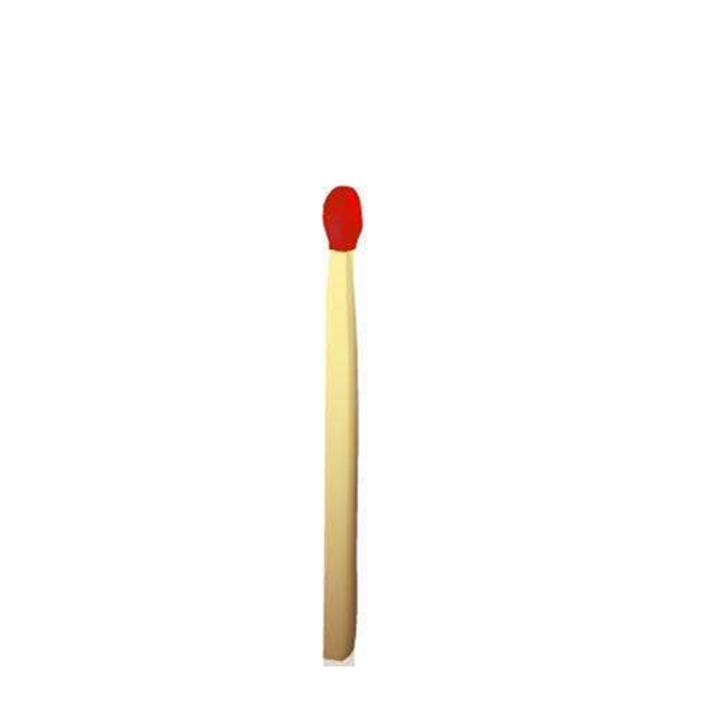

Supplement: Supplementary Figure 1 — Order diagram of event occurrence. Each set of cards included 3 cards (12 cm × 12 cm) which represented the early, middle and late states of the event. [file Data_Sheet_1.zip › Exp2new-Cards in sequence of events/═╝╞1⁄431.png]

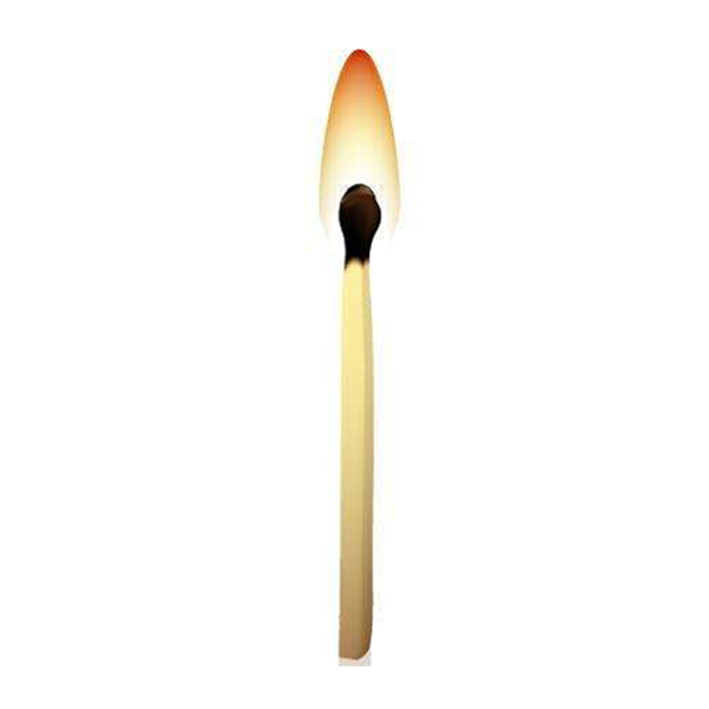

Supplement: Supplementary Figure 1 — Order diagram of event occurrence. Each set of cards included 3 cards (12 cm × 12 cm) which represented the early, middle and late states of the event. [file Data_Sheet_1.zip › Exp2new-Cards in sequence of events/═╝╞1⁄432.png]

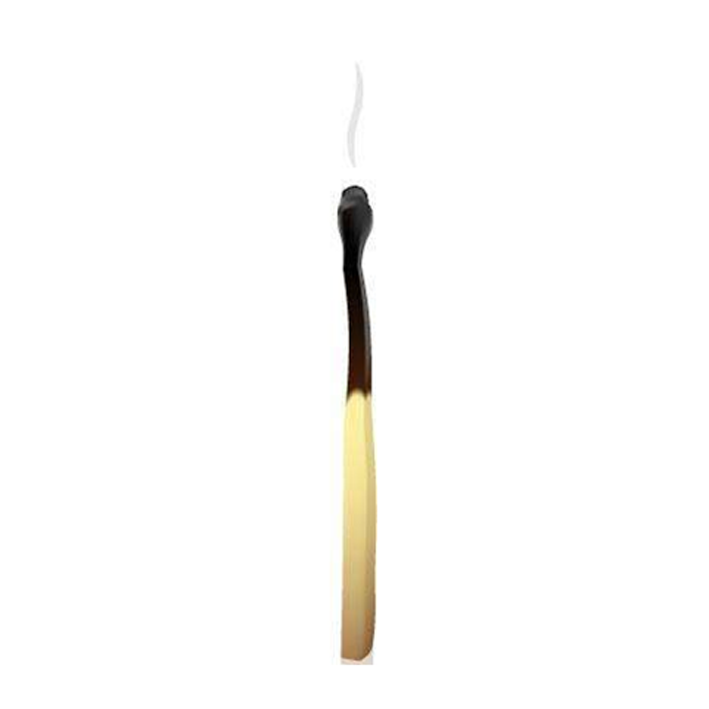

Supplement: Supplementary Figure 1 — Order diagram of event occurrence. Each set of cards included 3 cards (12 cm × 12 cm) which represented the early, middle and late states of the event. [file Data_Sheet_1.zip › Exp2new-Cards in sequence of events/═╝╞1⁄433.png]

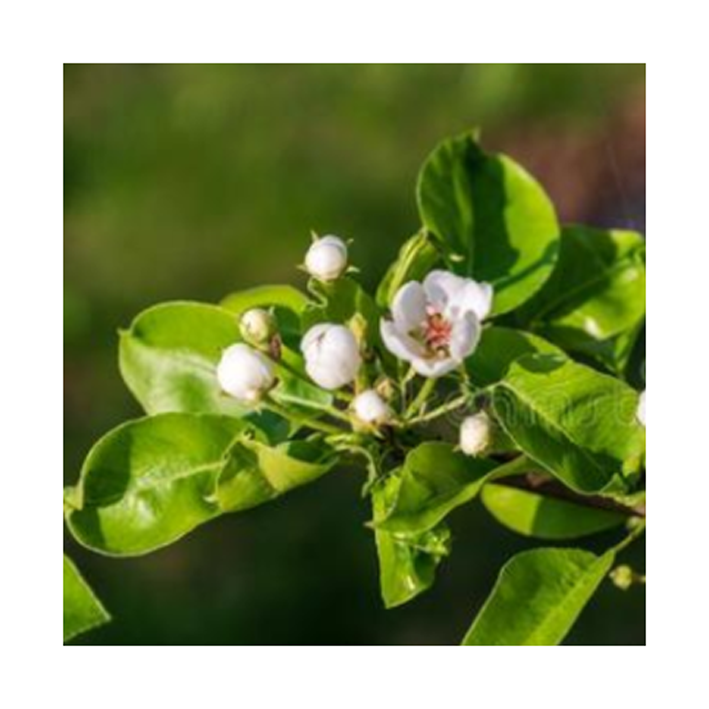

Supplement: Supplementary Figure 1 — Order diagram of event occurrence. Each set of cards included 3 cards (12 cm × 12 cm) which represented the early, middle and late states of the event. [file Data_Sheet_1.zip › Exp2new-Cards in sequence of events/═╝╞1⁄434.png]

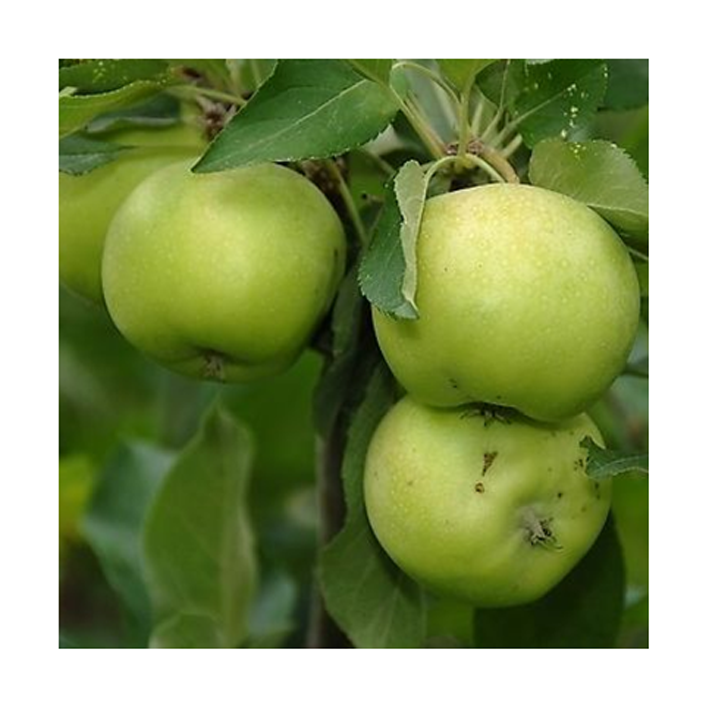

Supplement: Supplementary Figure 1 — Order diagram of event occurrence. Each set of cards included 3 cards (12 cm × 12 cm) which represented the early, middle and late states of the event. [file Data_Sheet_1.zip › Exp2new-Cards in sequence of events/═╝╞1⁄435.png]

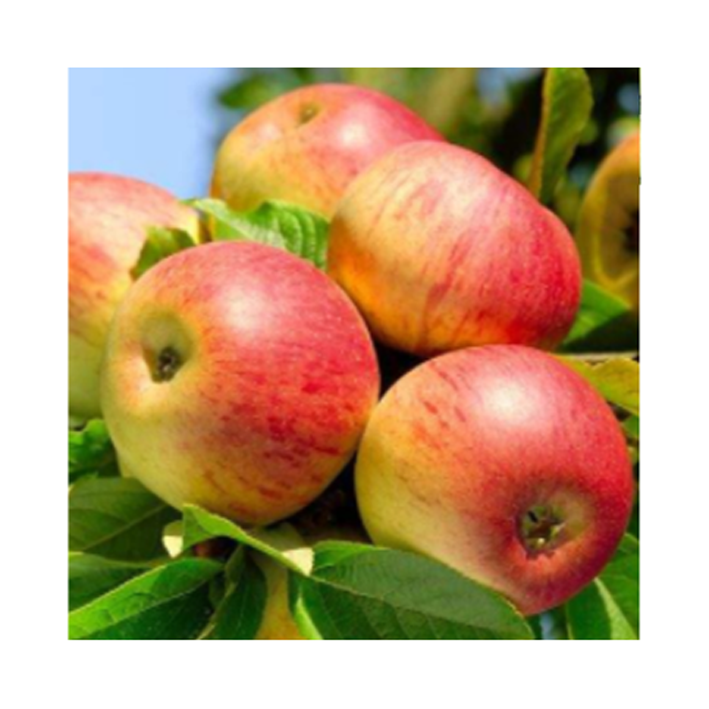

Supplement: Supplementary Figure 1 — Order diagram of event occurrence. Each set of cards included 3 cards (12 cm × 12 cm) which represented the early, middle and late states of the event. [file Data_Sheet_1.zip › Exp2new-Cards in sequence of events/═╝╞1⁄436.png]

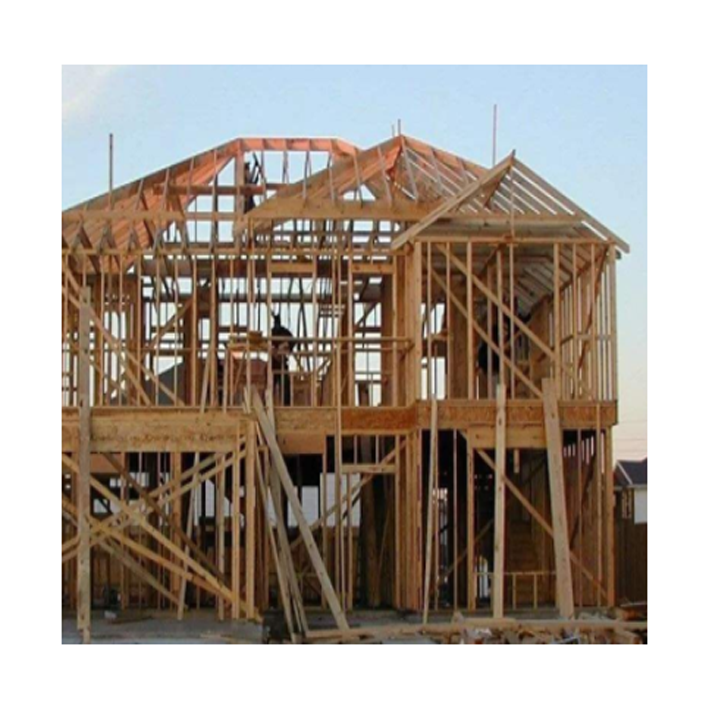

Supplement: Supplementary Figure 1 — Order diagram of event occurrence. Each set of cards included 3 cards (12 cm × 12 cm) which represented the early, middle and late states of the event. [file Data_Sheet_1.zip › Exp2new-Cards in sequence of events/═╝╞1⁄437.png]

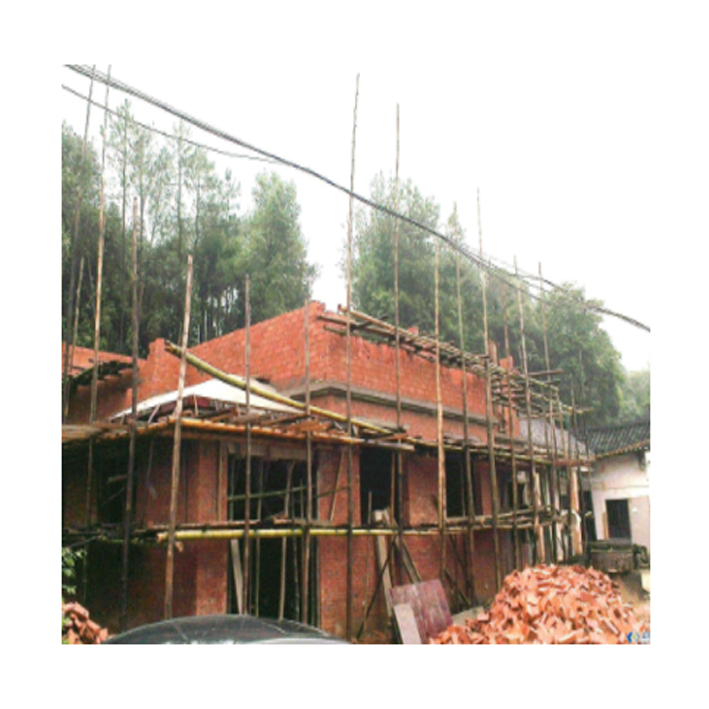

Supplement: Supplementary Figure 1 — Order diagram of event occurrence. Each set of cards included 3 cards (12 cm × 12 cm) which represented the early, middle and late states of the event. [file Data_Sheet_1.zip › Exp2new-Cards in sequence of events/═╝╞1⁄438.png]

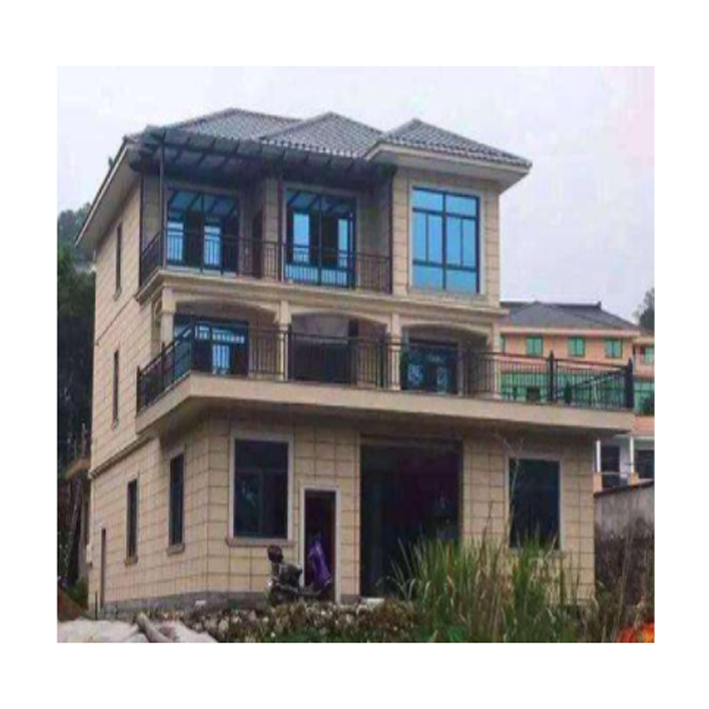

Supplement: Supplementary Figure 1 — Order diagram of event occurrence. Each set of cards included 3 cards (12 cm × 12 cm) which represented the early, middle and late states of the event. [file Data_Sheet_1.zip › Exp2new-Cards in sequence of events/═╝╞1⁄439.png]

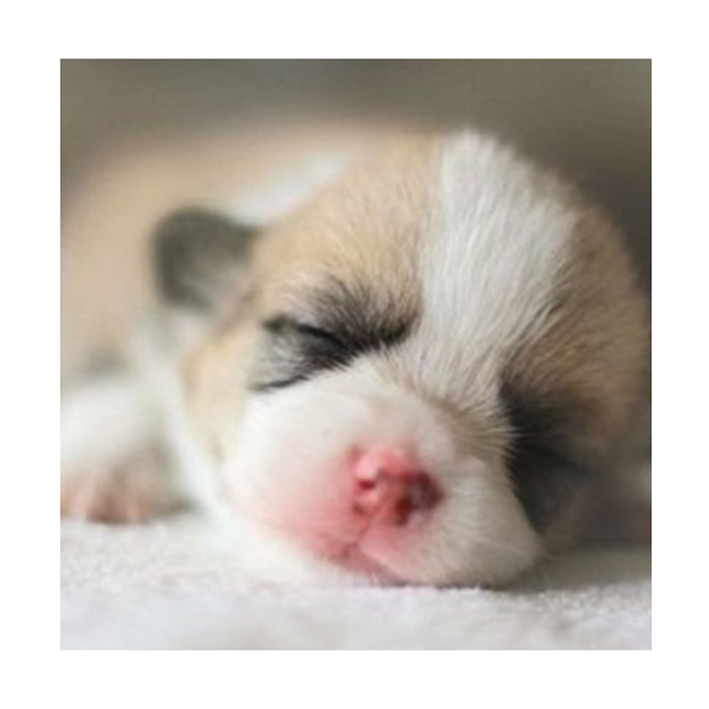

Supplement: Supplementary Figure 1 — Order diagram of event occurrence. Each set of cards included 3 cards (12 cm × 12 cm) which represented the early, middle and late states of the event. [file Data_Sheet_1.zip › Exp2new-Cards in sequence of events/═╝╞1⁄44.png]

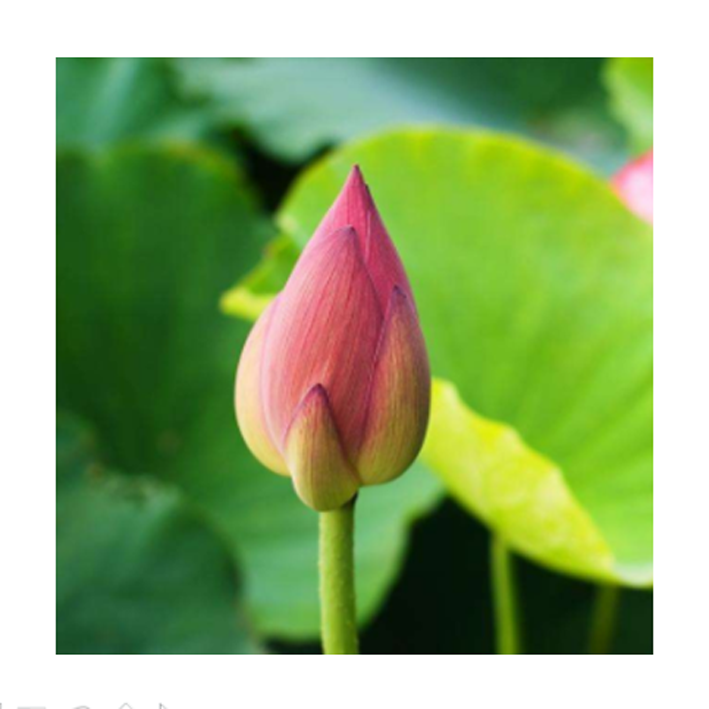

Supplement: Supplementary Figure 1 — Order diagram of event occurrence. Each set of cards included 3 cards (12 cm × 12 cm) which represented the early, middle and late states of the event. [file Data_Sheet_1.zip › Exp2new-Cards in sequence of events/═╝╞1⁄443.png]

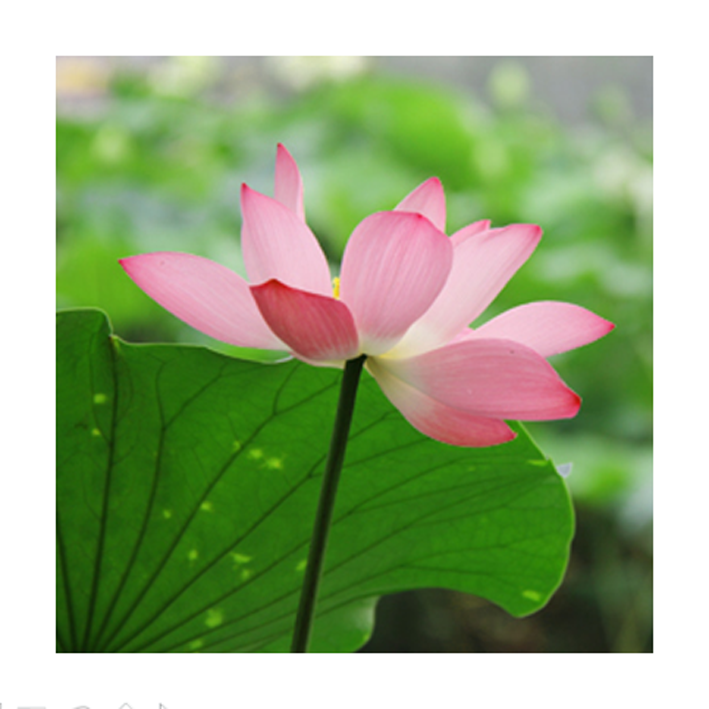

Supplement: Supplementary Figure 1 — Order diagram of event occurrence. Each set of cards included 3 cards (12 cm × 12 cm) which represented the early, middle and late states of the event. [file Data_Sheet_1.zip › Exp2new-Cards in sequence of events/═╝╞1⁄444.png]

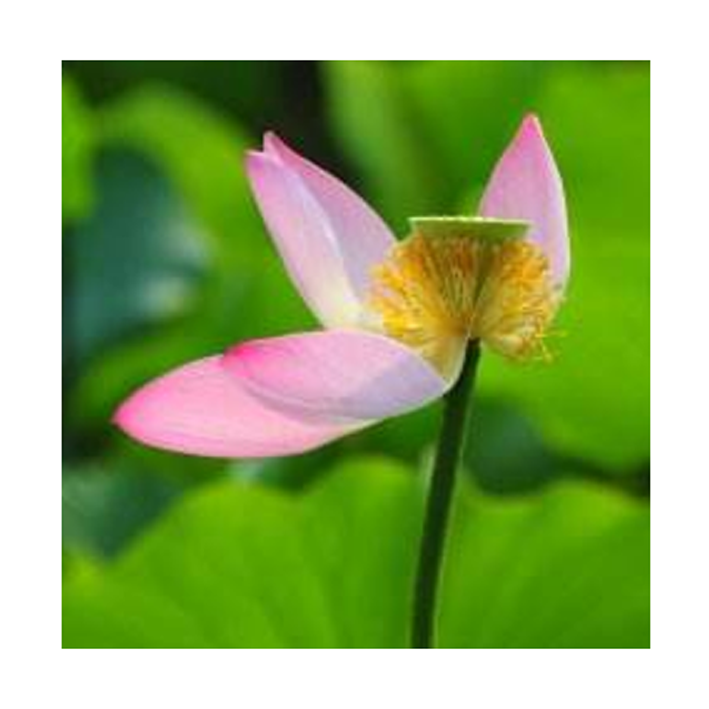

Supplement: Supplementary Figure 1 — Order diagram of event occurrence. Each set of cards included 3 cards (12 cm × 12 cm) which represented the early, middle and late states of the event. [file Data_Sheet_1.zip › Exp2new-Cards in sequence of events/═╝╞1⁄445.png]

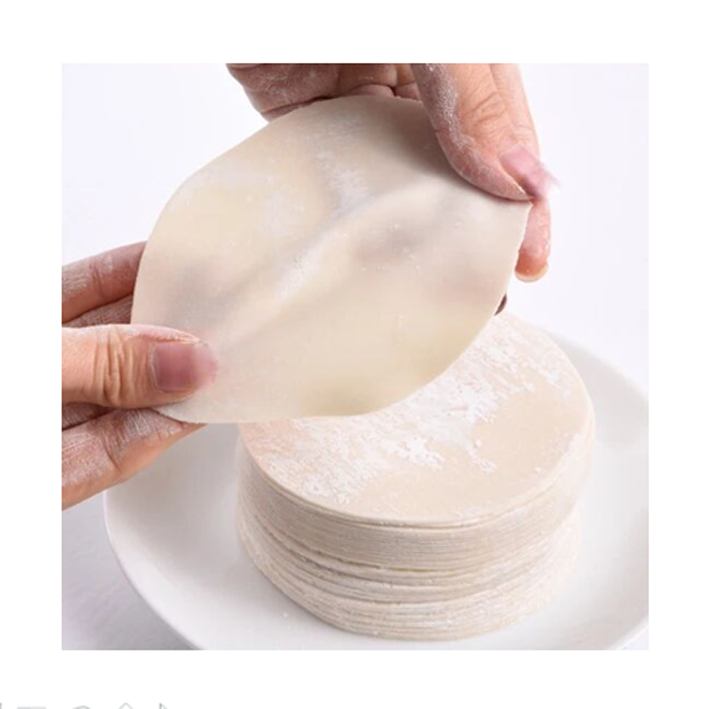

Supplement: Supplementary Figure 1 — Order diagram of event occurrence. Each set of cards included 3 cards (12 cm × 12 cm) which represented the early, middle and late states of the event. [file Data_Sheet_1.zip › Exp2new-Cards in sequence of events/═╝╞1⁄446.png]

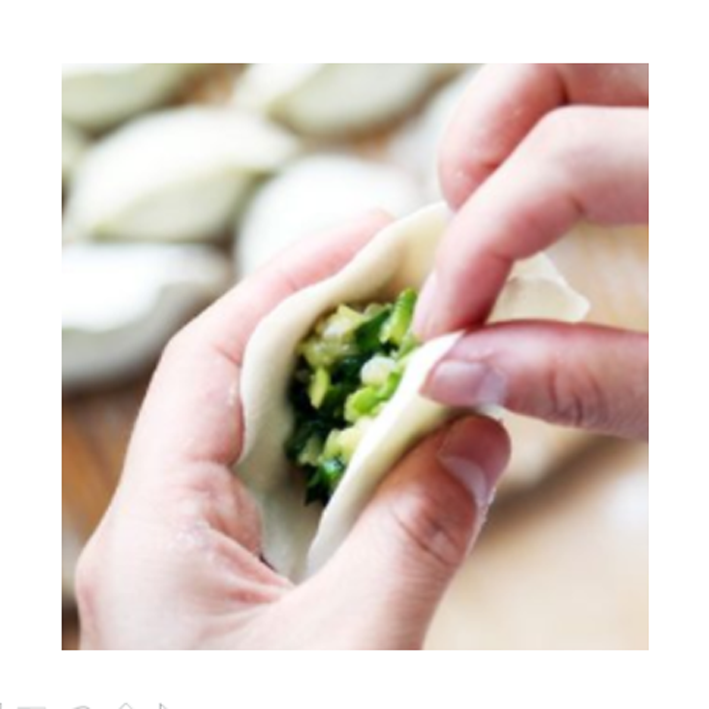

Supplement: Supplementary Figure 1 — Order diagram of event occurrence. Each set of cards included 3 cards (12 cm × 12 cm) which represented the early, middle and late states of the event. [file Data_Sheet_1.zip › Exp2new-Cards in sequence of events/═╝╞1⁄447.png]

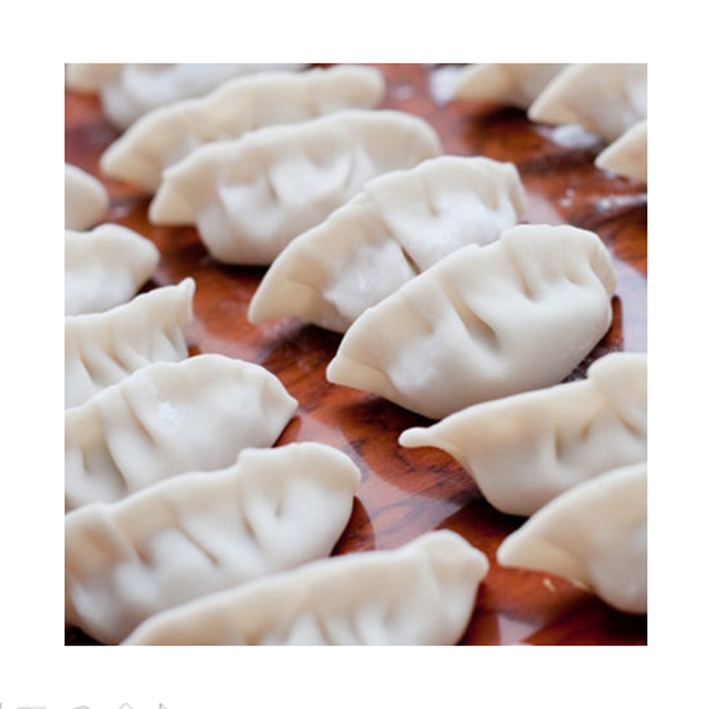

Supplement: Supplementary Figure 1 — Order diagram of event occurrence. Each set of cards included 3 cards (12 cm × 12 cm) which represented the early, middle and late states of the event. [file Data_Sheet_1.zip › Exp2new-Cards in sequence of events/═╝╞1⁄448.png]

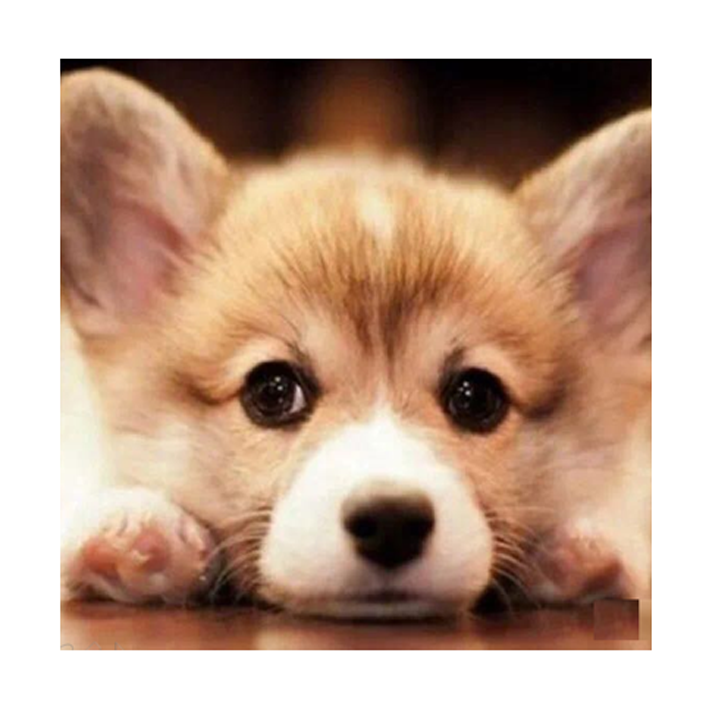

Supplement: Supplementary Figure 1 — Order diagram of event occurrence. Each set of cards included 3 cards (12 cm × 12 cm) which represented the early, middle and late states of the event. [file Data_Sheet_1.zip › Exp2new-Cards in sequence of events/═╝╞1⁄45.png]

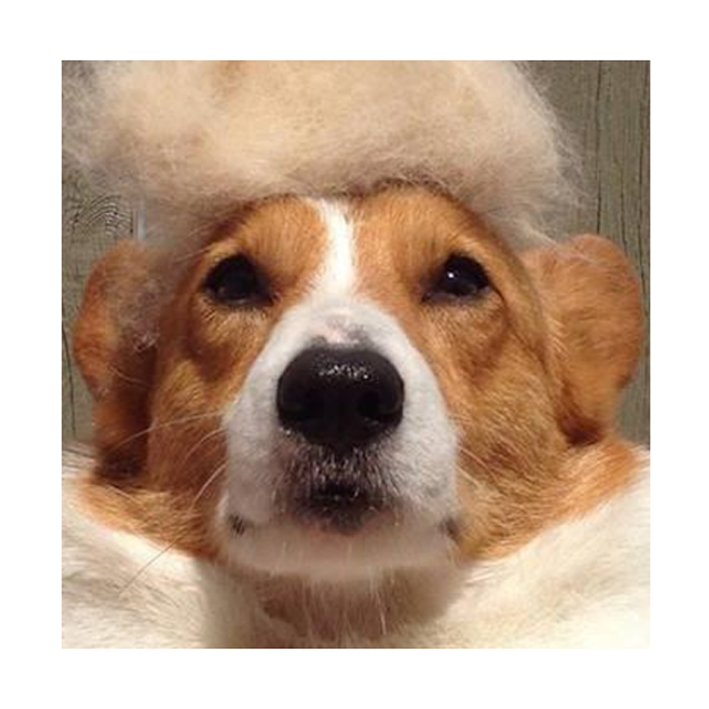

Supplement: Supplementary Figure 1 — Order diagram of event occurrence. Each set of cards included 3 cards (12 cm × 12 cm) which represented the early, middle and late states of the event. [file Data_Sheet_1.zip › Exp2new-Cards in sequence of events/═╝╞1⁄46.png]

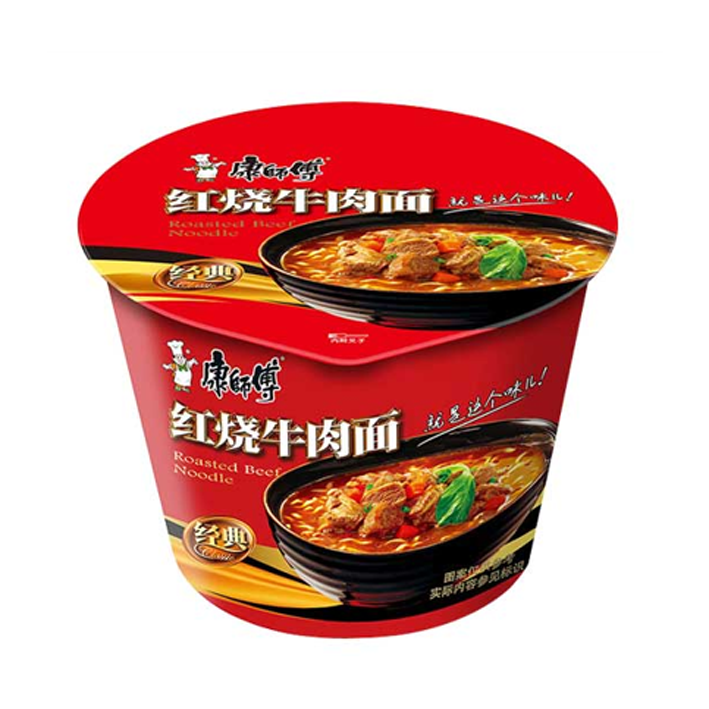

Supplement: Supplementary Figure 1 — Order diagram of event occurrence. Each set of cards included 3 cards (12 cm × 12 cm) which represented the early, middle and late states of the event. [file Data_Sheet_1.zip › Exp2new-Cards in sequence of events/═╝╞1⁄47.png]

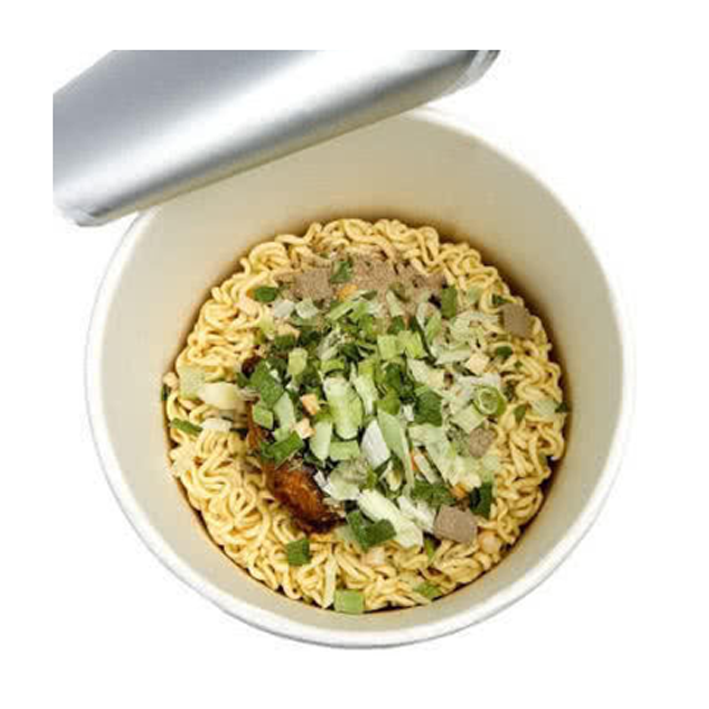

Supplement: Supplementary Figure 1 — Order diagram of event occurrence. Each set of cards included 3 cards (12 cm × 12 cm) which represented the early, middle and late states of the event. [file Data_Sheet_1.zip › Exp2new-Cards in sequence of events/═╝╞1⁄48.png]

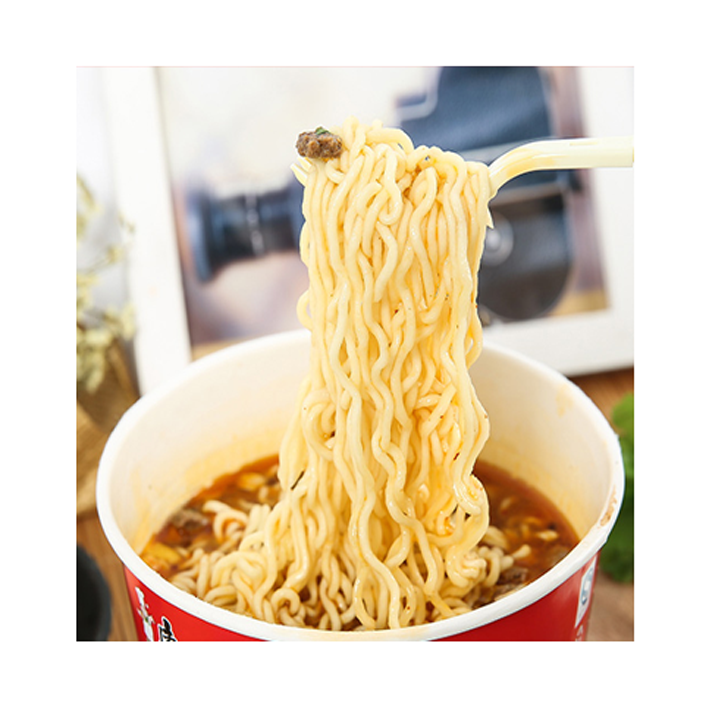

Supplement: Supplementary Figure 1 — Order diagram of event occurrence. Each set of cards included 3 cards (12 cm × 12 cm) which represented the early, middle and late states of the event. [file Data_Sheet_1.zip › Exp2new-Cards in sequence of events/═╝╞1⁄49.png]

|  | R | W | 123 | 321 | 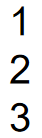 | 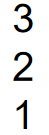 | 0  (other) |
| --- | --- | --- | --- | --- | --- | --- | --- |
| 1 |  |  |  |  |  |  |  |
| 2 |  |  |  |  |  |  |  |
| 3 |  |  |  |  |  |  |  |
| 4 |  |  |  |  |  |  |  |
| 5 |  |  |  |  |  |  |  |
| 6 |  |  |  |  |  |  |  |
| 7 |  |  |  |  |  |  |  |
| 8 |  |  |  |  |  |  |  |
| 9 |  |  |  |  |  |  |  |
| 10 |  |  |  |  |  |  |  |
| 11 |  |  |  |  |  |  |  |
| 12 |  |  |  |  |  |  |  |
| 13 |  |  |  |  |  |  |  |
| 14 |  |  |  |  |  |  |  |
| 15 |  |  |  |  |  |  |  |

Supplement: Supplementary Table 1 — Results Record Table of Card sorting task (Section). First record the judgment of the two small cards as “start” or “last”, correctly call “√” under “R” and “√” under “W”; then call “√” in the corresponding position in the table in the direction represented by the display itself. [file Table_1.DOC]

| Record 1 | | | Record 2 | | |
| --- | --- | --- | --- | --- | --- |
|  | 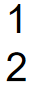 | 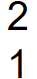 |  | 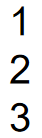 | 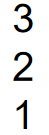 |
| 1 |  |  | 1 |  |  |
| 2 |  |  | 2 |  |  |
| 3 |  |  | 3 |  |  |
| 4 |  |  | 4 |  |  |
| 5 |  |  | 5 |  |  |
| 6 |  |  | 6 |  |  |
| 7 |  |  | 7 |  |  |
| 8 |  |  | 8 |  |  |
| 9 |  |  | 9 |  |  |
| 10 |  |  | 10 |  |  |
| 11 |  |  | 11 |  |  |
| 12 |  |  | 12 |  |  |
| 13 |  |  | 13 |  |  |
| 14 |  |  | 14 |  |  |
| 15 |  |  | 15 |  |  |

Supplement: Supplementary Table 2 — Record table of implicit and explicit task results (section). According to the placement results of the subject, play “√” in the corresponding position in the table according to the direction of the placement representative. [file Table_2.DOC]
